# Associations between colorectal cancer risk and dietary intake of tomato, tomato products, and lycopene: evidence from a prospective study of 101,680 US adults

**DOI:** 10.3389/fonc.2023.1220270

**Published:** 2023-08-11

**Authors:** Zongze Jiang, Huilin Chen, Ming Li, Wei Wang, Feiwu Long, Chuanwen Fan

**Affiliations:** ^1^ Department of Gastrointestinal, Bariatric and Metabolic Surgery, Research Center for Nutrition, Metabolism and Food Safety, West China-PUMC C.C. Chen Institute of Health, West China School of Public Health and West China Fourth Hospital, Sichuan University, Chengdu, China; ^2^ Department of Immunology, Institute of Basic Medical Sciences, Chinese Academy of Medical Sciences, Beijing, China; ^3^ School of Basic Medicine, Peking Union Medical College, Beijing, China; ^4^ Department of Nutrition, Food Hygiene, and Toxicology, West China School of Public Health and West China Fourth Hospital, Sichuan University, Chengdu, China; ^5^ Department of Oncology and Department of Biomedical and Clinical Sciences, Linköping University, Linköping, Sweden

**Keywords:** cohort, colorectal cancer, dietary nutrients, LYCOPENE, PLCO, tomato

## Abstract

**Background:**

Previous epidemiological studies have yielded inconsistent results regarding the effects of dietary tomato, tomato products, and lycopene on the incidence of colorectal cancer (CRC), possibly due to variations in sample sizes and study designs.

**Methods:**

The current study used multivariable Cox regression, subgroup analyses, and restricted cubic spline functions to investigate correlations between CRC incidence and mortality and raw tomato, tomato salsa, tomato juice, tomato catsup, and lycopene intake, as well as effect modifiers and nonlinear dose-response relationships in 101,680 US adults from the Prostate, Lung, Colorectal and Ovarian Cancer Screening Trial.

**Results:**

During follow-up 1100 CRC cases and 443 CRC-specific deaths occurred. After adjustment for confounding variables, high consumption of tomato salsa was significantly associated with a reduced risk of CRC incidence (hazard ratio comparing the highest category with the lowest category 0.8, 95% confidence interval 0.65–0.99, *p* for trend = 0.039), but not with a reduced risk of CRC mortality. Raw tomatoes, tomato juice, tomato catsup, and lycopene consumption were not significantly associated with CRC incidence or CRC mortality. No potential effect modifiers or nonlinear associations were detected, indicating the robustness of the results.

**Conclusion:**

In the general US population a higher intake of tomato salsa is associated with a lower CRC incidence, suggesting that tomato salsa consumption has beneficial effects in terms of cancer prevention, but caution is warranted when interpreting these findings. Further prospective studies are needed to evaluate its potential effects in other populations.

## 1 Introduction

Colorectal cancer (CRC) is a significant global public health challenge, and the third most prevalent cancer in the United States with an estimated 147,950 new cases and 53,200 deaths in 2020 (1). Unhealthy lifestyle factors including heavy alcohol consumption, cigarette smoking, physical inactivity, excess body weight, and dietary choices may contribute to nearly half of CRC cases (2). Emerging evidence suggests that high consumption of red or processed meat (3, 4), trans-fatty acids (5) and dietary supplements containing aristolochic acid (6) may increase the risk of CRC, whereas consumption of calcium (7, 8), whole grains and fiber (9), fruit and vegetables (10), and dairy products (11) may decrease the risk. It would therefore be beneficial to establish a primary prevention strategy after clarifying associations between different dietary components and CRC incidence.

Tomatoes and tomato products are recognized as a component of a healthy diet (12). Epidemiological studies have shown that higher intake of tomato, tomato products, and/or lycopene may reduce the risk of various cancers, including hepatocellular carcinoma (13), prostate cancer (14), pancreatic cancer (15), gastric cancer (16), and ovarian cancer (17). Nevertheless, associations between tomato/tomato product intake and CRC risk remain unclear due to limited participant sizes and inconsistent study results (18, 19). Meta-analyses have yielded conflicting results with regard to associations between lycopene intake and the incidence of CRC (20, 21). Notably, these studies did not differentiate between raw and processed tomatoes, which may have different effects on CRC risk. Dose-response relationships between tomato or lycopene intake and mortality have not been investigated. A recent study investigated relationships between the intake of raw tomatoes, tomato catsup, or lycopene and all-cause and cause-specific mortality, but no such analysis has been done to examine their relationship with CRC (22).

To provide evidence to fill this gap, we conducted a comprehensive, prospective cohort study using data from the Prostate, Lung, Colorectal and Ovarian (PLCO) Cancer Screening Trial, which was a multicenter randomized controlled study involving approximately 155,000 participants. The aim of the current study was to investigate potential correlations between the risk of CRC incidence and mortality and the consumption of tomatoes, tomato products, and lycopene. Additionally, we sought to examine the possible dose-response relationships and nonlinear associations between the intake of tomato products/lycopene and CRC risk. We also aimed to enhance the generalizability of our findings by conducting subgroup analyses.

## 2 Materials and methods

### 2.1 Data source and study population

The PLCO study design and methodology have been previously described (23). Briefly, it was a multicenter randomized controlled trial aimed at determining whether specific screening examinations reduce mortality from PLCO cancers. Approximately 155,000 participants aged 55–74 years were recruited between 1993 and 2001 *via* ten screening centers across the United States, and were randomly assigned to either a control group or an intervention group upon entry, in accordance with a detailed plan. The study was approved by the NCI’s Institutional Review Boards and each study center, and all enrolled participants signed informed consent forms.

**Figure 1:**
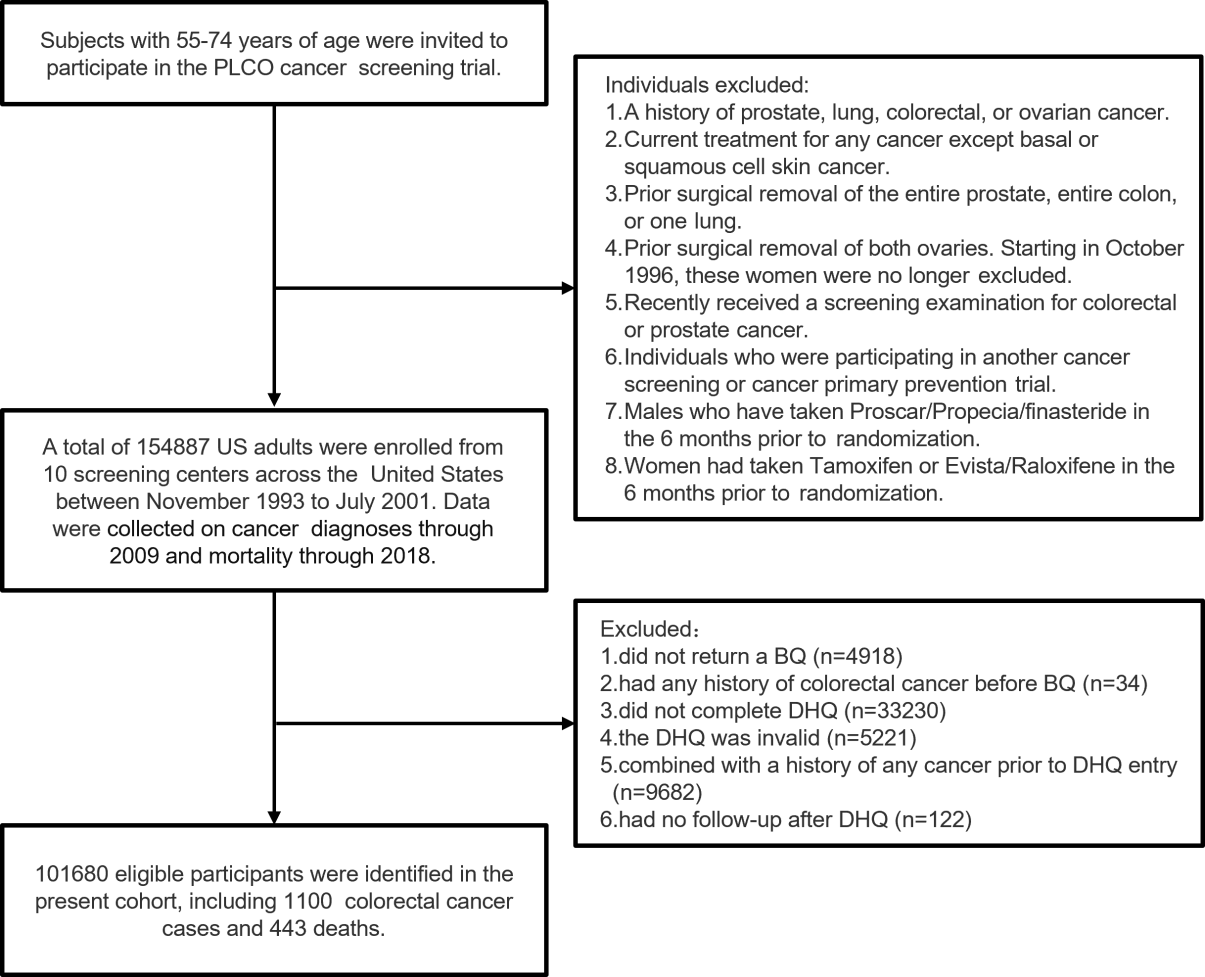
The flow chart of study participants from the PLCO screening trial.

Participants were excluded if they (1) did not return the baseline questionnaire (*n* = 4918) or had any history of CRC before the baseline questionnaire (*n* = 34) (2); had an incomplete dietary history questionnaire (DHQ) (*n* = 33,230) or an invalid DHQ that was missing the completion date, was completed before the date of death, had ≥ 8 missing frequency responses, or indicated extremely high or low calorie intake (*i.e.*, top 1% or bottom 1%) (*n* = 5,221) (3); had a history of any cancer before DHQ entry (*n* = 9,682); or (4) no follow-up time after the DHQ (*n* = 122). Ultimately 101,680 eligible participants were included in our cohort (**Figure 1**).

### 2.2 Data collection and dietary assessment

All participants completed a baseline questionnaire in which they self-reported information on demographics and medical history, including sex, race, trial arm, body mass index (BMI), educational level, marital status, aspirin use, cigarette smoking, family history of CRC, history of colon comorbidities, history of colorectal polyps, and diabetes history. Dietary data were collected using a self-administered DHQ. The DHQ included the serving size and response frequency of 124 food items and supplement use over the past year, such as red meat, processed meat, fruit, vegetables, whole grain, dairy, added sugars, dietary fiber, protein, total fat, carbohydrate, glycemic load, glycemic index, calcium, folate, magnesium, iron, vitamin D, and olive oil. The 1994-96 Continuing Survey of Food Intakes by Individuals, available from the USDA Food Surveys Research Group, and the Nutrition Data Systems for Research from the University of Minnesota were used to calculate the daily intake of all nutrients in the database (24). The DHQ has been validated and has shown good or better performance in estimating dietary intake compared to other commonly used food frequency questionnaires (25). Five independent exposures were included in the current analysis; tomato juice, raw tomato, tomato salsa, tomato catsup, and lycopene. Due to a lack of data on total tomato consumption, the overall relationship between total tomato and CRC risk could not be investigated.

### 2.3 Outcome ascertainment

The primary endpoint of the study was the incidence of CRC, which was determined *via* annual medical record reviews that updated participants’ cancer diagnosis status, including the date of detection and the site of the cancer. The secondary endpoint was mortality related to CRC. Information regarding deaths was obtained through various sources, including Annual Study Update questionnaires, reports from relatives, friends, or physicians, and National Death Index Plus searches. Upon notification, PLCO Screening Centers made efforts to obtain a death certificate for each death that occurred on or before 31 December 2018. The trial database recorded and coded information from the death certificate, and the underlying cause of death was determined using rules established by the National Center for Health Statistics. To ensure a more accurate assessment of trial endpoints a death review process was conducted, and medical records were reviewed for all deaths that may have been related to prostate, lung, colorectal, and ovarian cancers. The DRP cause of death was considered authoritative and was used in statistical analyses of the primary endpoints. The follow-up duration was calculated from the date of completion of the DHQ to the first occurrence of CRC diagnosis, participant dropout, CRC-related death, or the end of follow-up through to 31 December 2009 for incidence, and through to 31 December 2018 for mortality.

### 2.4 Statistical analyses

Dietary exposures were adjusted for total energy from the diet using the residual method (26). Energy-adjusted dietary tomato, tomato products, and lycopene intakes were then divided equally into quintiles, with the lowest quintile serving as the referent group. Continuous variables are expressed as medians and interquartile ranges (IQRs), and categorical variables are presented as numbers and percentages. Kruskal-Wallis H tests and chi-squared tests were used to compare between-group variance if appropriate. Multivariable Cox regression analyses were used to estimate hazard ratios (HRs) and 95% confidence intervals (CIs). Schoenfeld residuals were used to verify the proportional hazard assumption of baseline covariates (all *p* > 0.05) (27). Due to the abnormal distribution of the five exposures, a Log2 transformation was performed. The linear trend of each quintile of energy-adjusted dietary tomato, tomato products, and lycopene intakes were also analyzed by entering the median value as a continuous variable in the models. Model 2 was fully adjusted for age, sex, race, trial arm, BMI, educational level, marital status, aspirin use, cigarette smoking, alcohol consumption, family history of CRC, history of colon comorbidities, history of colorectal polyps, diabetes history, and dietary energy intake. In addition, the five exposures were mutually adjusted to assess individual contributions to the risk of CRC.

Subgroup analyses were conducted in several prespecified subgroups, including age group, sex, trial arm, BMI group, aspirin use, cigarette smoking, alcohol consumption, family history of CRC, history of colon comorbidities, colorectal polyps, and diabetes. The interaction effect on each stratum was compared using likelihood-ratio tests. Restricted cubic spline functions with four knots (5_th_, 35_th_, 65_th_, and 95_th_ percentiles) were used to investigate non-linear associations between dietary tomato, tomato product, and lycopene intakes and the incidence and CRC mortality. Notably subjects with energy-adjusted dietary tomato/lycopene intakes < 1_st_ or > 90_th_ percentile were excluded to reduce potential bias for extreme values in the dose-response analyses.

Several sensitivity analyses were conducted as follows: (1) Excluding events within the first 2 years of follow-up (1636 participants excluded); (2) excluding events involving extreme energy intake (< 800/> 4000 kcal/day for men and < 500/> 3500 kcal/day for women) (2886 participants excluded); (3) additional adjustment for the factors listed in the fully-adjusted model (model 2; **Table 1**), and processed meat (g/day), red meat (g/day), vegetables (g/day), fruit (g/day), whole grain (servings/day), sugar (tsp/day), dairy (servings/day), and dietary fiber (g/day); (4) additional adjustment for glycemic load, glycemic index, protein (% energy), total fat (% energy), and carbohydrate (% energy) in model 2; (5) additional adjustment for total calcium (mg/day), folate (mg/day), magnesium (mg/day), iron (mg/day), and vitamin D (µg/day) in model 3; (6) additional adjustment for olive oil (g/day) in model 4. Additional analyses to investigate associations between the five dietary exposures and CRC mortality were also conducted. All analyses were performed using R statistical software (http://www.R-project.org, R Foundation) and the Free Statistics analysis platform (28). All tests were two-tailed, and the significance level was set at 0.05.

**Table 1:** Baseline characteristics of study population according to quintiles of energy-adjusted tomato salsa consumption in 101680 participants.

		Quintiles of energy-adjusted tomato salsa consumption (g/day)					
Variables	Overall	Q1 (≤0.068)	Q2 (0.068 to 0.639)	Q3 (0.639 to 1.22)	Q4 (1.22 to 3.165)	Q5 (≥3.165)	p
Number of participants	101680	20336	20336	20336	20335	20337	
Age at DHQ (years)	65.0 (61.0, 70.0)	66.0 (61.0, 71.0)	66.0 (62.0, 71.0)	66.0 (61.0, 70.0)	64.0 (60.0, 69.0)	63.0 (60.0, 68.0)	< 0.001
Sex							< 0.001
Male	49441 (48.6)	14043 (69.1)	9641 (47.4)	7952 (39.1)	8317 (40.9)	9488 (46.7)	
Female	52239 (51.4)	6293 (30.9)	10695 (52.6)	12384 (60.9)	12019 (59.1)	10848 (53.3)	
Trial arm							0.049
Intervention	51767 (50.9)	10411 (51.2)	10288 (50.6)	10233 (50.3)	10322 (50.8)	10513 (51.7)	
Control	49913 (49.1)	9925 (48.8)	10048 (49.4)	10103 (49.7)	10014 (49.2)	9823 (48.3)	
Race							< 0.001
White, Non-Hispanic	92465 (91.0)	18112 (89.1)	18268 (89.8)	18124 (89.1)	18961 (93.3)	19000 (93.5)	
Black, Non-Hispanic	3352 (3.3)	1074 (5.3)	864 (4.2)	828 (4.1)	364 (1.8)	222 (1.1)	
Hispanic	1493 (1.5)	195 (1)	151 (0.7)	202 (1)	275 (1.4)	670 (3.3)	
Others	4333 (4.3)	947 (4.7)	1050 (5.2)	1177 (5.8)	731 (3.6)	428 (2.1)	
Missing	37 (0.0)	8 (0)	3 (0)	5 (0)	5 (0)	16 (0.1)	
Marital status							< 0.001
Married	79578 (78.3)	16048 (78.9)	15789 (77.6)	15494 (76.2)	16007 (78.7)	16240 (79.9)	
Unmarried	21916 (21.6)	4258 (20.9)	4501 (22.1)	4805 (23.6)	4287 (21.1)	4065 (20)	
Missing	186 (0.2)	30 (0.1)	46 (0.2)	37 (0.2)	42 (0.2)	31 (0.2)	
Education level							< 0.001
≤high school	42909 (42.2)	9704 (47.7)	9289 (45.7)	9055 (44.5)	7691 (37.8)	7170 (35.3)	
≥some college	58574 (57.6)	10598 (52.1)	10999 (54.1)	11242 (55.3)	12602 (62)	13133 (64.6)	
Missing	197 (0.2)	34 (0.2)	48 (0.2)	39 (0.2)	43 (0.2)	33 (0.2)	
BMI (kg/m2)							< 0.001
<25	34426 (33.9)	6103 (30)	7109 (35)	7352 (36.2)	7201 (35.4)	6661 (32.8)	
≥25	65915 (64.8)	13947 (68.6)	12935 (63.6)	12717 (62.5)	12875 (63.3)	13441 (66.1)	
Missing	1339 (1.3)	286 (1.4)	292 (1.4)	267 (1.3)	260 (1.3)	234 (1.2)	
Aspirin use							< 0.001
No	53472 (52.6)	10358 (50.9)	10741 (52.8)	10983 (54)	10759 (52.9)	10631 (52.3)	
Yes	47775 (47.0)	9896 (48.7)	9488 (46.7)	9254 (45.5)	9517 (46.8)	9620 (47.3)	
Missing	433 (0.4)	82 (0.4)	107 (0.5)	99 (0.5)	60 (0.3)	85 (0.4)	
Cigarette smoking							0.007
Never	48532 (47.7)	8955 (44.0)	10103 (49.7)	10243 (50.4)	9896 (48.7)	9335 (45.9)	
Current	9393 (9.2)	2570 (12.6)	1951 (9.6)	1855 (9.1)	1581 (7.8)	1436 (7.1)	
Former	43742 (43.0)	8807 (43.3)	8278 (40.7)	8237 (40.5)	8857 (43.6)	9563 (47)	
Missing	13 (0.0)	4 (0)	4 (0)	1 (0)	2 (0)	2 (0)	
Alcohol drinking							< 0.001
Never	10110 (10.2)	1842 (9.3)	2183 (11.1)	2351 (11.9)	1897 (9.6)	1837 (9.2)	
Former	14746 (14.9)	3410 (17.2)	3233 (16.5)	3100 (15.8)	2486 (12.5)	2517 (12.7)	
Current	73944 (74.8)	14525 (73.4)	14210 (72.4)	14230 (72.3)	15447 (77.9)	15532 (78.1)	
Missing	2880 (2.8)	559 (2.7)	710 (3.5)	655 (3.2)	506 (2.5)	450 (2.2)	
Family history of colorectal cancer							< 0.001
No	88113 (87.3)	17546 (87)	17475 (86.5)	17587 (87.2)	17724 (87.9)	17781 (88)	
Yes	10300 (10.2)	2028 (10.1)	2184 (10.8)	2094 (10.4)	2024 (10)	1970 (9.8)	
Possibly	2493 (2.5)	600 (3.0)	532 (2.6)	497 (2.5)	416 (2.1)	448 (2.2)	
Missing	774 (0.8)	162 (0.8)	145 (0.7)	158 (0.8)	172 (0.8)	137 (0.7)	
Diabetes							< 0.001
No	94353 (92.8)	18742 (92.2)	18781 (92.4)	18763 (92.3)	19029 (93.6)	19038 (93.6)	
Yes	6801 (6.7)	1489 (7.3)	1463 (7.2)	1465 (7.2)	1200 (5.9)	1184 (5.8)	
Missing	526 (0.5)	105 (0.5)	92 (0.5)	108 (0.5)	107 (0.5)	114 (0.6)	
History of colorectal polyps							< 0.001
No	94305 (92.7)	18704 (92)	18785 (92.4)	18890 (92.9)	18958 (93.2)	18968 (93.3)	
Yes	6762 (6.7)	1511 (7.4)	1438 (7.1)	1322 (6.5)	1255 (6.2)	1236 (6.1)	
Missing	613 (0.6)	121 (0.6)	113 (0.6)	124 (0.6)	123 (0.6)	132 (0.6)	
History of colon comorbidities							0.071
No	99439 (97.8)	19863 (97.7)	19880 (97.8)	19868 (97.7)	19889 (97.8)	19939 (98)	
Yes	1355 (1.3)	300 (1.5)	289 (1.4)	272 (1.3)	265 (1.3)	229 (1.1)	
Missing	886 (0.9)	173 (0.9)	167 (0.8)	196 (1.0)	182 (0.9)	168 (0.8)	
Total energy from diet (kcal/day)	1607 (1222, 2101)	2309 (1996, 2833)	1549 (1361, 1746)	1159 (966.8, 1619)	1425 (1035, 1758)	1598 (1247, 2150)	< 0.001
Red meat (g/day)	47.8 (26.8, 80.2)	80.3 (49.3, 123.4)	46.9 (28.0, 72.3)	34.4 (20.0, 56.9)	40.3 (23.4, 66.1)	48.2 (27.3, 81.0)	< 0.001
Processed meat (g/day)	10.8 (5.1, 22.5)	20.4 (9.8, 37.1)	10.9 (5.3, 21.3)	7.6 (3.8, 15.5)	8.8 (4.5, 17.6)	10.5 (5.1, 21.4)	< 0.001
Fruit (g/day)	231.6 (128.8, 359.6)	276.5 (150.6, 438.5)	239.3 (134.1, 362.1)	200.1 (110.2, 312.5)	211.4 (118.0, 329.9)	236.7 (136.0, 365.7)	< 0.001
Vegetables (g/day)	242.9 (159.1, 359.4)	302.6 (206.4, 439.0)	227.9 (153.5, 326.1)	187.2 (120.4, 281.4)	219.8 (144.0, 321.7)	290.0 (196.6, 417.3)	< 0.001
Whole grain (servings/day)	1.0 (0.6, 1.6)	1.3 (0.8, 2.0)	1.0 (0.6, 1.5)	0.8 (0.5, 1.3)	0.9 (0.5, 1.4)	1.0 (0.6, 1.6)	< 0.001
Dairy (servings/day)	1.1 (0.6, 1.8)	1.6 (0.9, 2.6)	1.1 (0.6, 1.8)	0.8 (0.4, 1.4)	0.9 (0.5, 1.5)	1.0 (0.6, 1.7)	< 0.001
Add sugars (tsp/day)	10.2 (6.6, 15.6)	16.7 (11.7, 23.9)	10.5 (7.5, 14.6)	7.8 (5.3, 11.6)	8.4 (5.4, 12.6)	9.4 (6.2, 14.2)	< 0.001
Dietary fiber (g/day)	16.5 (12.1, 22.2)	22.0 (17.3, 28.2)	16.0 (12.5, 20.3)	12.9 (9.5, 17.4)	14.8 (10.5, 19.8)	17.8 (13.2, 23.8)	< 0.001
Protein (% energy)	15.3 (13.5, 17.2)	15.0 (13.1, 16.9)	15.2 (13.4, 17.1)	15.2 (13.4, 17.2)	15.5 (13.7, 17.3)	15.6 (13.9, 17.5)	< 0.001
Total fat (% energy)	31.8 (26.6, 36.8)	33.6 (28.3, 38.3)	31.4 (26.3, 36.3)	30.7 (25.5, 35.7)	31.3 (26.4, 36.1)	32.2 (27.1, 37.1)	< 0.001
Carbohydrate (% energy)	52.0 (45.9, 58.1)	50.2 (44.5, 56.2)	53.0 (47.0, 59.0)	53.5 (47.4, 60.0)	52.1 (46.1, 58.0)	51.0 (45.0, 56.9)	< 0.001
Glycemic load	101.6 (76.7, 132.9)	146.6 (123.6, 178.4)	101.8 (86.7, 119.5)	78.2 (62.8, 101.8)	88.0 (64.2, 113.2)	98.3 (75.5, 130.6)	< 0.001
Glycemic index	53.6 (51.5, 55.7)	54.0 (51.9, 56.2)	53.9 (51.7, 56.0)	53.7 (51.5, 55.8)	53.4 (51.4, 55.4)	53.1 (51.1, 55.0)	< 0.001
Calcium (mg/day)	922.6 (600.9, 1337.0)	1113.0 (793.2, 1523.0)	888.8 (591.3, 1295.0)	765.2 (466.3, 1190.0)	859.0 (541.9, 1272.0)	967.1 (635.1, 1378.0)	< 0.001
Folate (mg/day)	593.5 (351.8, 755.5)	677.4 (436.0, 859.3)	591.0 (332.2, 738.0)	534.8 (275.7, 681.1)	568.0 (324.1, 723.8)	626.7 (387.8, 778.9)	< 0.001
Magnesium (mg/day)	354.0 (273.6, 446.4)	452.8 (372.7, 547.9)	345.9 (279.4, 414.9)	293.0 (223.0, 369.1)	323.1 (246.6, 407.6)	366.9 (288.1, 463.9)	< 0.001
Iron (mg/day)	24.0 (13.6, 31.7)	26.9 (17.7, 36.2)	22.4 (12.9, 31.2)	20.7 (10.4, 28.7)	23.3 (12.3, 30.3)	25.6 (14.6, 32.4)	< 0.001
Vitamin D (mcg/day)	10.8 (3.9, 13.5)	11.3 (5.1, 15.1)	10.7 (3.8, 13.6)	10.0 (3.0, 12.7)	10.8 (3.6, 13.1)	11.1 (4.0, 13.4)	< 0.001
Olive oil (g/day)	0.0 (0.0, 0.5)	0.0 (0.0, 0.3)	0.0 (0.0, 0.4)	0.0 (0.0, 0.3)	0.0 (0.0, 0.6)	0.0 (0.0, 0.8)	< 0.001

Data are presented as median (IQR) or number (percentage). “Others” refers to Asian, Pacific Islander, or American Indian. DHQ, dietary history of questionnaire; BMI, body mass index.

Energy from the diet was adjusted using the residual method.

## 3 Results

### 3.1 Participant characteristics

The cohort included 101,680 participants with a median follow-up of 9.54 years, corresponding to 908,801 person-years. During this period 1100 cases of CRC were reported, corresponding to an incidence rate of 12.10 per 10,000 person-years. Within a median follow-up of 14.5 years (corresponding to 1,353,326 person-years) 443 CRC-specific deaths were recorded. The average age of participants at baseline was 65.0 years. The median intakes of the five dietary items of primary interest were raw tomato 13.58 g/day, tomato salsa 0.90 g/day, tomato juice 12.76 g/day, tomato catsup 1.41 g/day, and lycopene 5.26 mg/day. The baseline characteristics of the study population according to the quintiles of the five exposure variables are summarized in **Table 1** and **Supplementary Tables S1**–**S4**. Compared to the lowest quintile of energy-adjusted tomato salsa consumption, participants in the highest quintile were more likely to be young (median age 64 years), Caucasian, more highly educated, have a history of diabetes, have a lower glycemic load, and have lower total dietary energy intake. On average they consumed less red meat, processed meat, and added sugars, and they were less likely to be current smokers. In the Q1 category (representing the lowest consumption of tomato salsa), 69.1% of participants were male. Overall, the distribution was similar however, with 48.6% being male and 51.4% being female. There were also similar trends in the consumption of other tomato products, including raw tomato, tomato juice, tomato ketchup, and lycopene (**Supplementary Tables S1**–**S4**).

### 3.2 Associations between CRC incidence and tomato, tomato product, and lycopene intakes

There was an inverse association between CRC incidence and the moderate and the highest dietary intake of tomato salsa and in the crude model (Q4 vs. Q1: HR 0.81, 95% CI 0.67–0.97; Q5 vs. Q1: HR 0.64, 95% CI 0.53–0.79) (**Table 2**). Similar results on the association between CRC incidence and the highest intake of tomato salsa were obtained in adjusted models (model 1, Q5 vs. Q1: HR 0.77, 95% CI 0.63–0.94, *p* trend = 0.016; model 2, HR Q5 vs. Q1: HR 0.80, 95% CI 0.65–0.99, *p* trend = 0.028). There were no significant associations between raw tomato, tomato juice, tomato catsup, or lycopene intake and CRC incidence. With respect to individual contributions to CRC incidence assessed after mutual adjustments for each of the five exposure variables, comparing tomato salsa Q5 and Q1 the HR for CRC incidence was 0.79 (95% CI 0.64–0.99, *p* = 0.037, *p* for trend = 0.030); thus tomato salsa intake remained a significant predictor of CRC risk even after adjustment for the other tomato variables and covariates.

**Table 2:** Association between energy-adjusted tomato-related products/lycopene intakes and colorectal cancer incidence in the PLCO cancer screening trial.

					HR (95%CI), *P*-value							
Variables	Cohort	Cases	Person-years	Incidence rate per 10,000 person-years	Crude model	*P*-value	Model 1	*P*-value	Model 2	*P*-value	Model 3	*P*-value
Tomato juice (g/day)												
Q1 (≤-19.516)	20336	223	181023.63	12.32	1(Ref)		1(Ref)		1(Ref)		1(Ref)	
Q2 (-19.516 to -14.884)	20336	210	182401.23	11.51	0.93 (0.77~1.13)	0.482	0.95 (0.79~1.15)	0.603	0.97 (0.8~1.19)	0.799	0.98 (0.8~	0.835
Q3 (-14.884 to -10.47)	20336	222	182438.83	12.17	0.99 (0.82~1.19)	0.898	1.02 (0.85~1.23)	0.836	1.05 (0.85~1.29)	0.652	1.06 (0.86	0.595
Q4 (-10.469 to -2.969)	20336	219	182517.78	12.00	0.97 (0.81~1.17)	0.784	1.01 (0.84~1.22)	0.901	1.04 (0.84~1.29)	0.73	1.05 (0.85	0.645
Q5 (≥-2.968)	20336	226	180419.57	12.53	1.02 (0.85~1.22)	0.86	1.01 (0.84~1.22)	0.877	1.03 (0.84~1.26)	0.778	1.03 (0.83	0.782
*P* for trend					0.727		0.673		0.612			0.59
continuous (log2)	101680	1100	908801.04	12.10	1.04 (0.98~1.09)	0.204	1.03 (0.97~1.09)	0.321	1.02 (0.96~1.08)	0.468	1.03 (0.96	0.476
Raw tomato (g/day)												
Q1 (≤5.221)	20336	218	179475.59	12.15	1(Ref)		1(Ref)		1(Ref)		1(Ref)	
Q2 (5.221 to 10.23)	20336	242	181125.62	13.36	1.1 (0.92~1.32)	0.306	1.11 (0.92~1.34)	0.262	1.14 (0.94~1.37)	0.19	1.14 (0.94	0.18
Q3 (10.23 to 17.767)	20336	231	182598.96	12.65	1.04 (0.87~1.25)	0.663	1.07 (0.89~1.29)	0.477	1.1 (0.91~1.33)	0.329	1.11 (0.91	0.306
Q4 (17.768 to 31.401)	20335	202	183210.42	11.03	0.91 (0.75~1.1)	0.325	0.93 (0.77~1.13)	0.474	0.96 (0.79~1.16)	0.658	0.96 (0.79	0.679
Q5 (≥31.402)	20337	207	182390.46	11.35	0.93 (0.77~1.13)	0.488	0.97 (0.8~1.18)	0.766	0.99 (0.81~1.2)	0.904	0.98 (0.8~	0.851
*P* for trend					0.128		0.273		0.355			0.341
continuous (log2)	101680	1100	908801.04	12.10	0.97 (0.93~1.01)	0.117	0.98 (0.94~1.02)	0.253	0.98 (0.94~1.02)	0.293	0.98 (0.94	0.28
Tomato salsa (g/day)												
Q1 (≤0.068)	20336	246	179986.93	13.67	1(Ref)		1(Ref)		1(Ref)		1(Ref)	
Q2 (0.068 to 0.639)	20336	236	181861.51	12.98	0.95 (0.79~1.14)	0.571	0.98 (0.82~1.17)	0.823	1.01 (0.83~1.23)	0.921	1.01 (0.83	0.942
Q3 (0.639 to 1.22)	20336	255	181476.59	14.05	1.03 (0.86~1.22)	0.756	1.11 (0.93~1.33)	0.249	1.15 (0.93~1.43)	0.182	1.15 (0.93	0.19
Q4 (1.22 to 3.165)	20336	202	182733.37	11.05	0.81 (0.67~0.97)	0.025	0.93 (0.77~1.12)	0.426	0.97 (0.79~1.2)	0.798	0.97 (0.78	0.786
Q5 (≥3.165)	20336	161	182742.63	8.81	0.64 (0.53~0.79)	<0.001	0.77 (0.63~0.94)	0.009	0.8 (0.65~0.99)	0.039	0.79 (0.64	0.037
*P* for trend					<0.001		0.016		0.028			0.03
continuous (log2)	101680	1100	908801.04	12.10	0.95 (0.92~0.98)	0.001	0.97 (0.94~1)	0.078	0.97 (0.94~1.01)	0.127	0.97 (0.94	0.129
Tomato catsup (g/day)												
Q1 (≤0.139)	20335	237	181659.51	13.05	1(Ref)		1(Ref)		1(Ref)		1(Ref)	
Q2 (0.14 to 1.039)	20337	198	183181.59	10.81	0.83 (0.69~1)	0.051	0.85 (0.7~1.02)	0.084	0.86 (0.7~1.05)	0.138	0.86 (0.7~	0.138
Q3 (1.039 to 1.798)	20336	230	182380.14	12.61	0.97 (0.81~1.16)	0.713	1.01 (0.84~1.21)	0.946	1.02 (0.83~1.26)	0.852	1.02 (0.82	0.861
Q4 (1.798 to 3.197)	20336	235	180984.45	12.98	0.99 (0.83~1.19)	0.956	1.03 (0.86~1.23)	0.766	1.03 (0.83~1.28)	0.787	1.03 (0.83	0.798
Q5 (≥3.197)	20336	200	180595.36	11.07	0.85 (0.7~1.02)	0.087	0.87 (0.72~1.05)	0.144	0.86 (0.7~1.05)	0.135	0.86 (0.7~	0.131
*P* for trend					0.492		0.69		0.5			0.497
continuous (log2)	101680	1100	908801.04	12.10	0.99 (0.95~1.03)	0.613	0.99 (0.95~1.03)	0.719	0.99 (0.95~1.03)	0.502	0.98 (0.94	0.374
Lycopene (mcg/day)												
Q1 (≤3483.129)	20336	228	179571.41	12.70	1(Ref)		1(Ref)		1(Ref)		1(Ref)	
Q2 (3483.133 to 4716.331)	20336	220	182061.80	12.08	0.95 (0.79~1.15)	0.603	1 (0.83~1.2)	0.971	1.01 (0.83~1.22)	0.923	1.05 (0.87	0.61
Q3 (4716.353 to 5867.565)	20336	223	182526.27	12.22	0.96 (0.8~1.16)	0.686	1.05 (0.87~1.26)	0.615	1.06 (0.87~1.29)	0.548	1.13 (0.93	0.227
Q4 (5867.603 to 7913.297)	20336	215	183271.34	11.73	0.92 (0.77~1.11)	0.409	1.04 (0.86~1.26)	0.685	1.04 (0.86~1.27)	0.663	1.15 (0.93	0.192
Q5 (≥7913.508)	20336	214	181370.22	11.80	0.93 (0.77~1.12)	0.442	1.01 (0.84~1.22)	0.896	1.01 (0.84~1.22)	0.918	1.15 (0.92	0.229
*P* for trend					0.409		0.753		0.818			0.149
continuous (log2)	101680	1100	908801.04	12.10	0.95 (0.89~1.01)	0.092	0.98 (0.92~1.04)	0.407	0.97 (0.92~1.03)	0.391	1 (0.93~1.	0.914

PLCO, prostate, lung, colorectal and ovarian; HR, hazard ratio; CI, confidence interval.

Crude model adjusted for none.

Model 1 adjusted for age (continuous), sex (male vs. female), trial arm (intervention vs. control), and race (white, non-Hispanic vs. black, non-Hispanic vs. Hispanic vs. others).

Model 2 adjusted for model 1 plus marital status (married vs. unmarried), education level (≤high school vs. ≥some college), aspirin use (no vs. yes), diabetes (no vs. yes), cigarette smoking (never vs. current vs. former), BMI (<25kg/m2 vs. ≥25kg/m2), family history of colorectal cancer (yes vs. no vs. possibly), alcohol drinking (never vs. former vs. current), history of colorectal polyps (no vs. yes), history of colon comorbidities (no vs. yes), and energy from diet (continuous).

Model 3 adjusted for model 2, and mutually adjusted for the five exposure variables. Missing values for covariates were treated as dummy variables in the models.

There were no significant interactions between tomato salsa intake and CRC incidence in any subgroups including age, sex, trial arm, BMI group, aspirin use, cigarette smoking, alcohol drinking, family history of CRC, history of colon comorbidities, colorectal polyps, and diabetes (**Supplementary Table S5**, *p* for interaction > 0.05). Given the distinct distribution of tomato salsa intake between males and females within the Q1 category, we also performed subgroup analyses to assess the association between tomato salsa intake (as quintiles) and CRC risk by sex. There was a negative association between tomato salsa intake and CRC incidence in women (Q5 vs. Q1 HR 0.60, 95% CI 0.41–0.87, *p* = 0.007). In men there was only a tendency towards a negative association. There was no significant interaction effect between sex and salsa intake on CRC incidence (*p* for interaction = 0.703).

Sensitivity analyses were performed to examine the robustness of the correlation between tomato salsa intake and CRC incidence. The analyses included the exclusion of events ascertained within 2 years, the exclusion of subjects with extreme energy intakes, and the use of additional models. In those analyses the correlation between tomato salsa intake and CRC incidence remained robust (**Supplementary Table S6**). Smooth curve-fitting plots did not provide any evidence of nonlinear dose-response associations between energy-adjusted tomato salsa consumption and CRC incidence after full adjustment (**Supplementary Figure S1**; *p* for nonlinearity > 0.05).

### 3.3 Associations between CRC-specific mortality and tomato, tomato product, and lycopene intakes

Consumption of tomato salsa was significantly associated with lower CRC-specific mortality in the crude model (**Table 3**, *p* trend = 0.024). After adjustment for confounding variables in models 1 and 2 however, there were no significant associations (**Table 3**, *p* trend > 0.05). There were no significant associations between CRC-specific mortality and the intake of raw tomato, tomato juice, tomato ketchup, or lycopene. When the five exposures were mutually adjusted, comparing tomato salsa Q5 and Q1 yielded an HR for CRC mortality of 0.96 (95% CI 0.7–1.32, *p* = 0.807, *p* for trend = 0.942). Thus, there was no significant association between tomato salsa intake and CRC mortality.

**Table 3:** Association between energy-adjusted tomato-related products/lycopene intakes and colorectal cancer mortality in the PLCO cancer screening trial.

					HR (95%CI), *P*-value							
Variables	Cohort	Cases	Person-years	Mortality rate per 10,000 person-years	Crude model	*P*-value	Model 1	*P*-value	Model 2	*P*-value	Model 3	*P*-value
Tomato juice (g/day)												
Q1 (≤-19.516)	20336.00	93	270367.28	3.44	1(Ref)		1(Ref)		1(Ref)		1(Ref)	
Q2 (-19.516 to -14.884)	20336.00	88	272108.64	3.23	0.94 (0.7~1.26)	0.677	0.97 (0.72~1.3)	0.819	1.05 (0.77~1.44)	0.763	1.04 (0.76~1.43)	0.802
Q3 (-14.884 to -10.47)	20336.00	88	272483.46	3.23	0.94 (0.7~1.26)	0.669	0.99 (0.73~1.33)	0.929	1.09 (0.78~1.51)	0.61	1.08 (0.77~1.49)	0.663
Q4 (-10.469 to -2.969)	20336.00	82	272262.61	3.01	0.88 (0.65~1.18)	0.38	0.93 (0.69~1.25)	0.619	1.02 (0.73~1.44)	0.895	1.01 (0.72~1.42)	0.96
Q5 (≥-2.968)	20336.00	92	266104.28	3.46	1.01 (0.76~1.35)	0.947	1.02 (0.76~1.36)	0.913	1.08 (0.79~1.47)	0.644	1.02 (0.73~1.42)	0.915
*P* for trend						0.881		0.989		0.746		0.981
continuous (log2)	101680	443	1353326.28	3.27	1.06 (0.98~1.16)	0.164	1.05 (0.96~1.14)	0.295	1.03 (0.94~1.13)	0.519	1.01 (0.91~1.13)	0.805
Raw tomato (g/day)												
Q1 (≤5.221)	20336.00	86	266774.04	3.22	1(Ref)		1(Ref)		1(Ref)		1(Ref)	
Q2 (5.221 to 10.23)	20336.00	93	269208.28	3.45	1.07 (0.8~1.43)	0.655	1.09 (0.81~1.46)	0.568	1.18 (0.87~1.6)	0.284	1.16 (0.86~1.58)	0.328
Q3 (10.23 to 17.767)	20336.00	100	272218.88	3.67	1.13 (0.85~1.51)	0.394	1.18 (0.88~1.58)	0.259	1.28 (0.95~1.73)	0.101	1.26 (0.93~1.69)	0.136
Q4 (17.768 to 31.401)	20335.00	90	272689.76	3.30	1.02 (0.76~1.37)	0.906	1.06 (0.79~1.43)	0.706	1.15 (0.85~1.55)	0.377	1.11 (0.82~1.51)	0.509
Q5 (≥31.402)	20337.00	74	272435.32	2.72	0.84 (0.61~1.14)	0.266	0.89 (0.65~1.22)	0.458	0.93 (0.68~1.28)	0.659	0.87 (0.63~1.21)	0.402
*P* for trend						0.266		0.468		0.648		0.405
continuous (log2)	101680	443	1353326.28	3.27	0.98 (0.92~1.05)	0.624	1 (0.93~1.06)	0.881	1 (0.94~1.06)	0.961	0.99 (0.92~1.05)	0.656
Tomato salsa (g/day)												
Q1 (≤0.068)	20336	108	264446.75	4.08	1(Ref)		1(Ref)		1(Ref)		1(Ref)	
Q2 (0.068 to 0.639)	20336	79	268031.53	2.95	0.72 (0.54~0.96)	0.027	0.75 (0.56~1.01)	0.056	0.8 (0.58~1.1)	0.166	0.79 (0.58~1.09)	0.154
Q3 (0.639 to 1.22)	20336	100	269777.20	3.71	0.91 (0.69~1.19)	0.473	1 (0.76~1.33)	0.977	1.08 (0.78~1.5)	0.647	1.07 (0.77~1.48)	0.687
Q4 (1.22 to 3.165)	20336	73	275162.64	2.65	0.65 (0.48~0.87)	0.004	0.77 (0.57~1.04)	0.092	0.84 (0.6~1.17)	0.302	0.83 (0.59~1.16)	0.283
Q5 (≥3.165)	20336	83	275908.16	3.01	0.73 (0.55~0.97)	0.032	0.92 (0.69~1.23)	0.558	0.98 (0.72~1.33)	0.896	0.96 (0.7~1.32)	0.807
*P* for trend						0.024		0.614		0.97		0.942
continuous (log2)	101680	443	1353326.28	3.27	1 (0.95~1.06)	0.901	1.03 (0.98~1.09)	0.203	1.03 (0.98~1.09)	0.202	1.03 (0.98~1.09)	0.229
Tomato catsup (g/day)												
Q1 (≤0.139)	20335	96	271019.18	3.54	1(Ref)		1(Ref)		1(Ref)		1(Ref)	
Q2 (0.14 to 1.039)	20337	75	272550.36	2.75	0.78 (0.57~1.05)	0.101	0.81 (0.59~1.09)	0.163	0.9 (0.65~1.25)	0.532	0.89 (0.64~1.24)	0.495
Q3 (1.039 to 1.798)	20336	93	271560.51	3.42	0.97 (0.73~1.29)	0.823	1.03 (0.77~1.38)	0.822	1.2 (0.86~1.67)	0.282	1.18 (0.85~1.64)	0.324
Q4 (1.798 to 3.197)	20336	100	269238.67	3.71	1.05 (0.8~1.39)	0.717	1.11 (0.84~1.47)	0.469	1.29 (0.93~1.8)	0.132	1.27 (0.91~1.77)	0.168
Q5 (≥3.197)	20336	79	268957.56	2.94	0.83 (0.62~1.12)	0.231	0.86 (0.64~1.16)	0.32	0.92 (0.67~1.26)	0.612	0.89 (0.65~1.23)	0.481
*P* for trend						0.861		0.951		0.757		0.913
continuous (log2)	101680	443	1353326.28	3.27	1.01 (0.94~1.07)	0.859	1.01 (0.95~1.08)	0.774	1 (0.94~1.07)	0.968	0.99 (0.93~1.06)	0.832
Lycopene (mcg/day)												
Q1 (≤3483.129)	20336	89	265708.55	3.35	1(Ref)		1(Ref)		1(Ref)		1(Ref)	
Q2 (3483.133 to 4716.331)	20336	102	270495.69	3.77	1.12 (0.84~1.49)	0.427	1.2 (0.9~1.59)	0.222	1.26 (0.94~1.7)	0.124	1.3 (0.97~1.76)	0.082
Q3 (4716.353 to 5867.565)	20336	77	273019.15	2.82	0.84 (0.62~1.14)	0.253	0.94 (0.69~1.28)	0.684	1 (0.72~1.37)	0.985	1.05 (0.76~1.46)	0.768
Q4 (5867.603 to 7913.297)	20336	74	274401.73	2.70	0.8 (0.59~1.09)	0.156	0.93 (0.68~1.27)	0.652	0.97 (0.71~1.35)	0.876	1.05 (0.75~1.47)	0.764
Q5 (≥7913.508)	20336	101	269701.15	3.74	1.11 (0.84~1.48)	0.457	1.25 (0.94~1.66)	0.133	1.26 (0.94~1.68)	0.121	1.38 (0.99~1.94)	0.058
*P* for trend						0.767		0.518		0.495		0.292
continuous (log2)	101680	443	1353326.28	3.27	0.96 (0.87~1.05)	0.347	0.99 (0.9~1.09)	0.827	0.99 (0.9~1.09)	0.852	1 (0.89~1.12)	0.955

PLCO, prostate, lung, colorectal and ovarian; HR, hazard ratio; CI, confidence interval.

Crude model adjusted for none.

Model 1 adjusted for age (continuous), sex (male vs. female), trial arm (intervention vs. control), and race (white, non-Hispanic vs. black, non-Hispanic vs. Hispanic vs. others).

Model 2 adjusted for model 1 plus marital status (married vs. unmarried), education level (≤high school vs. ≥some college), aspirin use (no vs. yes), diabetes (no vs. yes), cigarette smoking (never vs. current vs. former), BMI (<25kg/m2 vs. ≥25kg/m2), family history of colorectal cancer (yes vs. no vs. possibly), alcohol drinking (never vs. former vs. current), history of colorectal polyps (no vs. yes), history of colon comorbidities (no vs. yes), and energy from diet (continuous).

Mode 3 adjusted mode 2, and mutually adjusted the five-exposure variables. Missing values for covariates were treated as dummy variables in the models.

In subgroup analyses there were no significant effect modifiers in the prespecified groups when the exposures were treated as categorical variables (quintiles) (**Supplementary Table S7**; *p* for interaction > 0.05). In sensitivity analyses there was also a lack of an association between dietary tomato salsa intake and CRC mortality (**Supplementary Table S8**). In dose-response analyses there was no non-linear relationship between tomato salsa intake and CRC mortality (**Supplementary Figures S2**; *p* for non-linearity > 0.05).

## 4 Discussion

In this prospective cohort study of 101,680 US adults’ higher consumption of tomato salsa was associated with a 20% lower risk of CRC incidence after adjustment for potential confounders. There were no significant associations between the consumption of raw tomato, tomato juice, tomato catsup, or lycopene and the risk of CRC incidence or mortality. These results were robust in a series of analyses. No effect modifiers or no non-linear relationships were observed. To our knowledge this is the first study to report a protective effect of tomato salsa against CRC risk. In contrast, a previous study did not observe a significant association between bladder cancer risk and tomato salsa consumption after adjustment for confounders in the PLCO cohort (29). These results suggest that the protective effect of tomato salsa against cancer risk is heterogenous among different cancers. To further assess the individual contribution of tomato-related dietary intake and CRC risk, we conducted a series of analyses including mutual adjustment for the five primary dietary factors of interest, and additional adjustment for other foods and nutrients, and the association between tomato salsa and CRC incidence remained robust. Although intake of tomato and/or lycopene has been associated with reduced risk of several cancers such as hepatocellular carcinoma (13), prostate cancer (14), pancreatic cancer (15), gastric cancer (16), ovarian cancer (17), and CRC (18, 19), in this large PLCO study CRC risk was not significantly associated with consumption of raw tomato, tomato juice, or tomato catsup. That was consistent with a previous study investigating bladder cancer (29), but inconsistent with a previous case-control study conducted in a CRC population in Italy, in which there was a protective association between a higher intake of tomato and the incidence of CRC (18), and sub-sites of CRC stratified by cancer site (19). These differences may be due to the retrospective nature of the previous studies on this topic, which only examined associations with total tomato intake. The current study investigated the effects of specific types of tomato products (*i.e.*, raw tomato and tomato catsup) on the incidence of CRC, which may have differential effects on health outcomes (29). Similarly, selection and recall bias due to the retrospective designs and residual confounders in previous studies may also have led the inconsistent results.

Although we did not specifically investigate the mechanisms underlying associations identified in the study, several potential explanations could be explored. It has been proposed that the cancer-preventing effects of high tomato consumption may be attributed to lycopene. This powerful antioxidant not only neutralizes harmful free radicals but also potentially mitigates oxidative stress, a condition associated with cellular damage and implicated in various types of cancer (30–32). Processed and concentrated tomato products such as salsa (9.28 mg/100 g) and tomato juice (7.83 mg/100 mg) contain higher levels of lycopene than raw tomatoes (3.1–7.74 mg/100 g) (33), which may contribute to their cancer-protective effects (34). However, we did not observe a significant association between CRC risk and dietary intake of lycopene after adjusting for confounders, which was similar to previously reported results (21, 35, 36). Notably our study only investigated the link between dietary lycopene intake and CRC risk, and did not directly measure serum lycopene levels. Because the estimated dietary lycopene absorption rate in humans ranges from 10%–30% (37), dietary intake may not fully reflect serum lycopene levels. Therefore, the observed correlational coefficient of 0.46 between dietary and serum lycopene levels could be influenced by various factors (14). In addition, the method of cooking and chopping can affect the bioavailability of lycopene, and certain food preparation techniques may enhance absorption (38, 39).

Tomato salsa (sofrito) is a traditional Mediterranean diet preparation comprised of a mix of foods characteristic of the Mediterranean diet such as tomato, onion, garlic, and extra virgin olive oil, and it contains many bioactive phenolic compounds and carotenoids (40, 41). The inverse association between salsa and CRC in our study may be attributable to the presence of unique additives such as olive oil. Olive oil is known to contain a variety of substances, including monounsaturated free fatty acids (such as oleic acid), hydrocarbon squalene, tocopherols, aroma components, and phenolic compounds. Although olive oil quality can affect biological/nutritional actions (42), these components have been associated with anticancer properties (43, 44). Therefore, the addition of olive oil to tomato sauces may have positive health outcomes. Furthermore, the potential health benefits may be related to the method of tomato salsa processing. Evidence from a prospective randomized, cross-over intervention study suggested that the plasma concentration and urinary excretion of naringenin glucuronide were both significantly higher after the consumption of tomato sauce than after the consumption of raw tomatoes. It was suggested that mechanical and thermal treatments during tomato sauce manufacture may help to deliver these potentially bioactive phenolics from the food matrix more effectively than the addition of an oil component, thus increasing their bioavailability (45). Moreover, tomato salsa, characterized by its intricate mixture of ingredients, should be considered for the potential synergistic interactions among its bioactive compounds. It is not only a rich source of antioxidant lycopene, but also a treasure trove of other bioactive components, including phenolic acids, flavonoids, and ascorbic acid (46). The interaction among these ingredients yields synergistic effects that amplify the health benefits of tomato salsa. For instance, the bioavailability of lycopene can be significantly boosted by the presence of fats, such as those found in avocados, olive oil - a common ingredient in salsa recipes (47). The assortment of antioxidants in salsa promises more robust protection against oxidative stress and inflammation than any single compound could offer (42). Lastly, participants with a higher intake of salsa often reported other healthy dietary habits at baseline, such as a higher intake of vegetables and fruits and a lower intake of red or processed meat, which may provide additional protective effects against CRC. To better understand the potential association between tomato and CRC risk, future studies should investigate the sources, bioavailability, and serum concentration of lycopene in tomato salsa, ideally with a longer follow-up period.

The strengths of this study included its prospective design based on a large and well-established cohort (the PLCO trial), which ensured reliable data, a large sample size, and a comprehensive assessment of dietary intake of various tomato products and lycopene. The data enabled investigation of dose-response relationships, as well as long-term follow-up with a high follow-up rate to minimize reverse causality and selection bias. The study also had several limitations. Firstly, due to the observational nature of the study there may have been residual confounders that we could not control for. Secondly, the data were derived from a dietary questionnaire, and may thus have been subject to recall bias and misclassification errors. Thirdly, we only had baseline dietary information, which limited our ability to examine dynamic changes between nutrients and cancer risk. Fourthly, serum assessment of nutrients was lacking, which prevented a more detailed evaluation. Fifthly, the study population was limited to the US, which may limit the generalizability of the results to other countries with different dietary patterns. Further studies with larger sample sizes and longer follow-up periods, as well as more detailed assessments of dietary intake and serum nutrient levels are warranted to confirm our findings and better understand potential associations between dietary factors and cancer risk.

## 5 Conclusions

The current study indicates that high amounts of tomato salsa may be a beneficial addition to a healthy diet, and may contribute to CRC prevention in the adult population in the US. However, more prospective studies that involve more detailed assessments of tomato salsa intake are necessary to assess its potential effects in other populations.

## Data availability statement

The original contributions presented in the study are included in the article/**Supplementary Material**. Further inquiries can be directed to the corresponding author.

## Ethics statement

As this study used data from the PLCO trial, which had obtained ethical permission, no additional ethical permit was required for our analysis. The patients/participants provided their written informed consent to participate in this study.

## Author contributions

Conceptualization: ZJ and HC. Methodology: ZJ and HC. Formal analysis: ZJ, WW, and ML. Original draft preparation: ZJ, HC, and CF. Supervision and project administration: ML, CF, and FL. Acquisition of data: ZJ and FL. Review and editing: ZJ, FL, and CF. All authors have read and agreed to the published version of the manuscript.
