# Supplementary material for: Associations between colorectal cancer risk and dietary intake of tomato, tomato products, and lycopene: evidence from a prospective study of 101,680 US adults
Source: Front Oncol. 2023 Aug 11;13:1220270. doi: 10.3389/fonc.2023.1220270 (PMC10457118; doi:10.3389/fonc.2023.1220270)
Supplement: Supplementary file 1 [file DataSheet_1.docx]

**Supplementary materials**

**Supplementary Figure S1**. Does-response analyses for the associations between energy-adjusted tomato-related products/lycopene intakes in colorectal incidence.

**Supplementary Figure S2**. Does-response analyses for the associations between energy-adjusted tomato-related products/lycopene intakes in colorectal cancer-specific mortality.

**Supplementary Table S1.** Baseline characteristics of study population according to quintiles of energy-adjusted raw tomato consumption in 101680 participants.

**Supplementary Table S2.** Baseline characteristics of study population according to quintiles of energy-adjusted tomato juice consumption in 101680 participants.

**Supplementary Table S3.** Baseline characteristics of study population according to quintiles of energy-adjusted tomato catsup consumption in 101680 participants.

**Supplementary Table S4.** Baseline characteristics of study population according to quintiles of energy-adjusted lycopene intake in 101680 participants.

**Supplementary Table S5.** Subgroup analyses of the associations between energy-adjusted tomato-related products/lycopene intakes (Quintiles) and colorectal cancer incidence.

**Supplementary Table S6.** Sensitivity analyses on the association between energy-adjusted tomato-related products consumption/ lycopene intakes and colorectal cancer incidence.

**Supplementary Table S7.** Subgroup analyses of the associations between energy-adjusted tomato-related products/lycopene intakes (Quintiles) and colorectal cancer mortality.

**Supplementary Table S8.** Sensitivity analyses on the association between energy-adjusted tomato-related products/ lycopene intakes and colorectal cancer mortality.


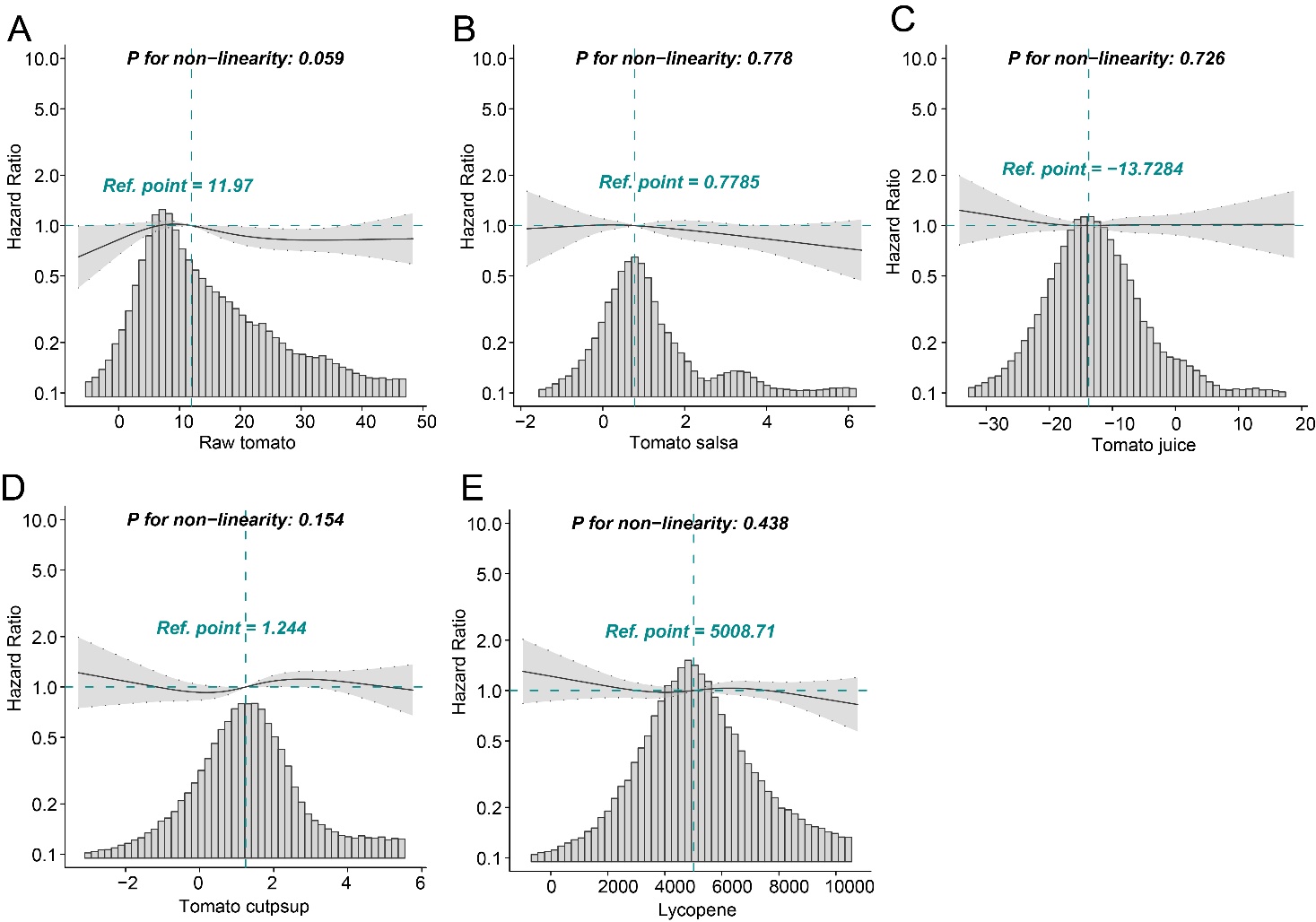


**Supplementary Figure S1**. Does-response analyses for the associations between energy-adjusted tomato-related products/lycopene intakes in colorectal incidence. Does-response analyses for the associations between energy-adjusted tomato juice (A), raw tomato (B), tomato salsa (C), tomato catsup (D), and lycopene (E) intakes and the risk of colorectal cancer using the restricted cubic spline model. Solid lines and the dashed lines represent the hazard ratios and corresponding 95% confidence intervals, which were calculated using restricted cubic spline regression with four knots (i.e., 5^th^, 35^th^, 65^th^, and 95^th^ percentiles) adjusting for age (continuous), sex (male vs. female), trial arm (intervention vs. control), and race (white, non-Hispanic vs. black, non-Hispanic vs. Hispanic vs. others), marital status (married vs. unmarried), education level (≤high school vs. ≥some college), aspirin use (no vs. yes), diabetes (no vs. yes), cigarette smoking (never vs. current vs. former), BMI (<25kg/m^2^ vs. ≥25kg/m^2^), family history of colorectal cancer (yes vs. no vs. possibly), alcohol drinking (never vs. former vs. current), history of colorectal polyps (no vs. yes), history of colon comorbidities (no vs. yes), and energy from the diet (continuous). Missing values for covariates were treated as dummy variables in the models.


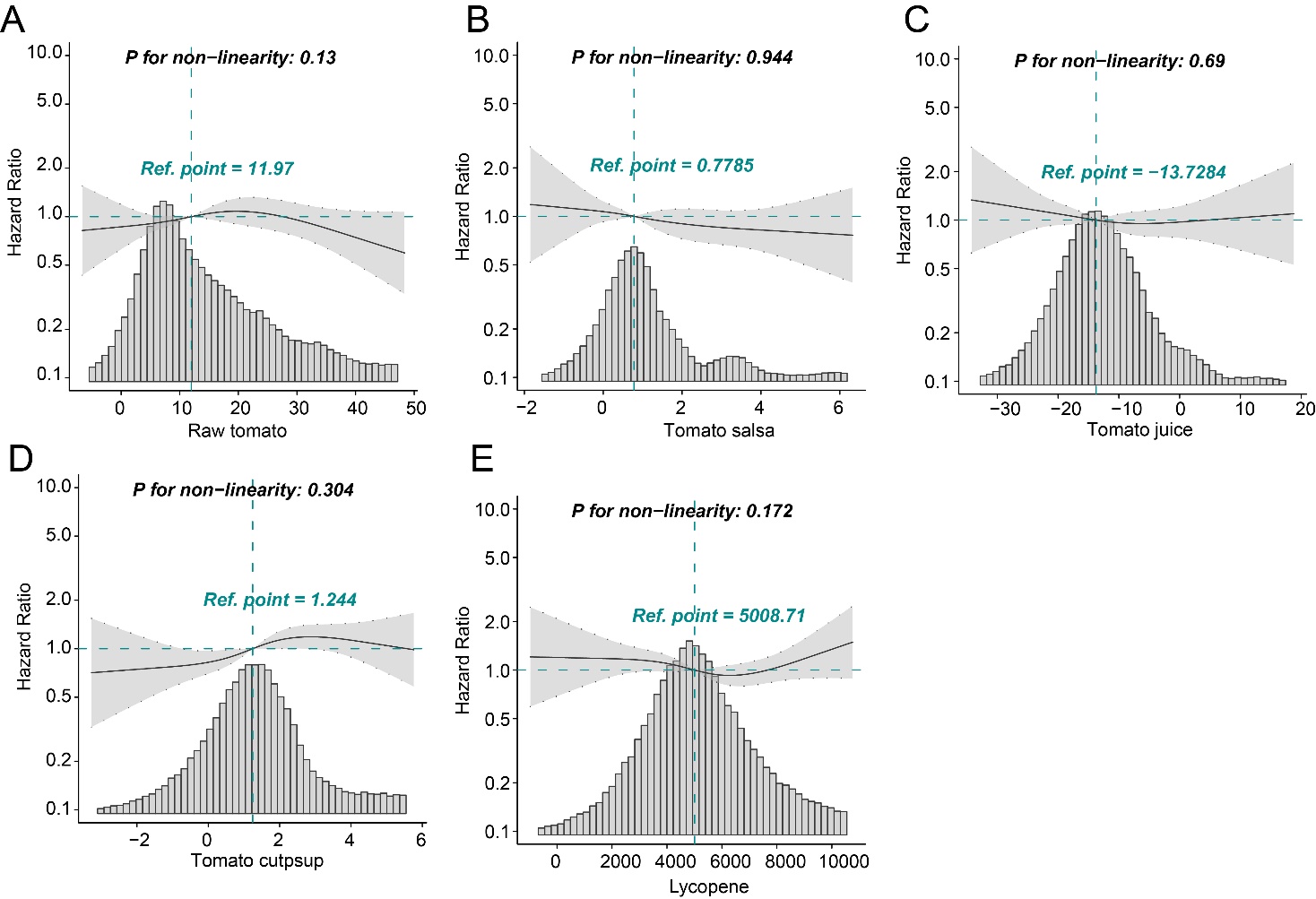


**Supplementary Figure S2**. Does-response analyses for the associations between energy-adjusted tomato-related products/lycopene intakes in colorectal cancer-specific mortality. Does-response analyses for the associations between energy-adjusted tomato juice (A), raw tomato (B), tomato salsa (C), tomato catsup (D), and lycopene (E) intakes and the risk of colorectal cancer using the restricted cubic spline model. Solid lines and the dashed lines represent the hazard ratios and corresponding 95% confidence intervals, which were calculated using restricted cubic spline regression with four knots (i.e., 5^th^, 35^th^, 65^th^, and 95^th^ percentiles) adjusting for age (continuous), sex (male vs. female), trial arm (intervention vs. control), and race (white, non-Hispanic vs. black, non-Hispanic vs. Hispanic vs. others), marital status (married vs. unmarried), education level (≤high school vs. ≥some college), aspirin use (no vs. yes), diabetes (no vs. yes), cigarette smoking (never vs. current vs. former), BMI (<25kg/m^2^ vs. ≥25kg/m^2^), family history of colorectal cancer (yes vs. no vs. possibly), alcohol drinking (never vs. former vs. current), history of colorectal polyps (no vs. yes), history of colon comorbidities (no vs. yes), and energy from the diet (continuous). Missing values for covariates were treated as dummy variables in the models.

| **Supplementary Table S1. Baseline characteristics of study population according to quintiles of energy-adjusted raw tomato consumption in 101680 participants.** | | | | | | | |  |
| --- | --- | --- | --- | --- | --- | --- | --- | --- |
|  |  | Quintiles of energy-adjusted raw tomato consumption (g/day) | | | | | |  |
| Variables | Overall | Q1 (≤5.221) | Q2 (5.221 to 10.23) | Q3 (10.23 to 17.767) | Q4 (17.768 to 31.401) | Q5 (≥31.402) | *p* |  |
| Number of participants | 101680 | 20336 | 20336 | 20336 | 20335 | 20337 |  |  |
| Age at DHQ (years) | 65.0 (61.0, 70.0) | 64.0 (60.0, 69.0) | 65.0 (61.0, 70.0) | 65.0 (61.0, 70.0) | 65.0 (61.0, 70.0) | 65.0 (61.0, 70.0) | < 0.001 |  |
| Sex |  |  |  |  |  |  | < 0.001 |  |
| Male | 49441 (48.6) | 13644 (67.1) | 9889 (48.6) | 8865 (43.6) | 8382 (41.2) | 8661 (42.6) |  |  |
| Female | 52239 (51.4) | 6692 (32.9) | 10447 (51.4) | 11471 (56.4) | 11953 (58.8) | 11676 (57.4) |  |  |
| Trial arm |  |  |  |  |  |  | 0.644 |  |
| Intervention | 51767 (50.9) | 10269 (50.5) | 10345 (50.9) | 10356 (50.9) | 10371 (51) | 10426 (51.3) |  |  |
| Control | 49913 (49.1) | 10067 (49.5) | 9991 (49.1) | 9980 (49.1) | 9964 (49) | 9911 (48.7) |  |  |
| Race |  |  |  |  |  |  | < 0.001 |  |
| White, Non-Hispanic | 92465 (91.0) | 17931 (88.2) | 18021 (88.7) | 18751 (92.2) | 18879 (92.9) | 18883 (92.9) |  |  |
| Black, Non-Hispanic | 3352 (3.3) | 991 (4.9) | 879 (4.3) | 575 (2.8) | 456 (2.2) | 451 (2.2) |  |  |
| Hispanic | 1493 (1.5) | 337 (1.7) | 300 (1.5) | 273 (1.3) | 261 (1.3) | 322 (1.6) |  |  |
| Others | 4333 (4.3) | 1070 (5.3) | 1128 (5.5) | 729 (3.6) | 733 (3.6) | 673 (3.3) |  |  |
| Missing | 37 (0.0) | 7 (0) | 8 (0) | 8 (0) | 6 (0) | 8 (0) |  |  |
| Marital status |  |  |  |  |  |  | < 0.001 |  |
| Married | 79578 (78.3) | 15562 (76.5) | 15537 (76.4) | 16074 (79) | 16306 (80.2) | 16099 (79.2) |  |  |
| Unmarried | 21916 (21.6) | 4736 (23.3) | 4750 (23.4) | 4224 (20.8) | 3994 (19.6) | 4212 (20.7) |  |  |
| Missing | 186 (0.2) | 38 (0.2) | 49 (0.2) | 38 (0.2) | 35 (0.2) | 26 (0.1) |  |  |
| Education level |  |  |  |  |  |  | < 0.001 |  |
| ≤high school | 42909 (42.2) | 9059 (44.5) | 8733 (42.9) | 8317 (40.9) | 8406 (41.3) | 8394 (41.3) |  |  |
| ≥some college | 58574 (57.6) | 11235 (55.2) | 11559 (56.8) | 11973 (58.9) | 11894 (58.5) | 11913 (58.6) |  |  |
| Missing | 197 (0.2) | 42 (0.2) | 44 (0.2) | 46 (0.2) | 35 (0.2) | 30 (0.1) |  |  |
| BMI (kg/m2) |  |  |  |  |  |  | < 0.001 |  |
| <25 | 34426 (33.9) | 6312 (31) | 6987 (34.4) | 7159 (35.2) | 7113 (35) | 6855 (33.7) |  |  |
| ≥25 | 65915 (64.8) | 13725 (67.5) | 13078 (64.3) | 12924 (63.6) | 12972 (63.8) | 13216 (65) |  |  |
| Missing | 1339 (1.3) | 299 (1.5) | 271 (1.3) | 253 (1.2) | 250 (1.2) | 266 (1.3) |  |  |
| Aspirin use |  |  |  |  |  |  | < 0.001 |  |
| No | 53472 (52.6) | 10415 (51.2) | 10910 (53.6) | 10776 (53) | 10761 (52.9) | 10610 (52.2) |  |  |
| Yes | 47775 (47.0) | 9821 (48.3) | 9310 (45.8) | 9487 (46.7) | 9489 (46.7) | 9668 (47.5) |  |  |
| Missing | 433 (0.4) | 100 (0.5) | 116 (0.6) | 73 (0.4) | 85 (0.4) | 59 (0.3) |  |  |
| Cigarette smoking |  |  |  |  |  |  | < 0.001 |  |
| Never | 48532 (47.7) | 8625 (42.4) | 9921 (48.8) | 10122 (49.8) | 10066 (49.5) | 9798 (48.2) |  |  |
| Current | 9393 (9.2) | 2538 (12.5) | 1896 (9.3) | 1711 (8.4) | 1614 (7.9) | 1634 (8) |  |  |
| Former | 43742 (43.0) | 9170 (45.1) | 8518 (41.9) | 8497 (41.8) | 8654 (42.6) | 8903 (43.8) |  |  |
| Missing | 13 (0.0) | 3 (0) | 1 (0) | 6 (0) | 1 (0) | 2 (0) |  |  |
| Alcohol drinking |  |  |  |  |  |  | < 0.001 |  |
| Never | 10110 (10.2) | 1657 (8.4) | 2066 (10.5) | 2039 (10.3) | 2122 (10.8) | 2226 (11.3) |  |  |
| Former | 14746 (14.9) | 3309 (16.7) | 3048 (15.4) | 2779 (14) | 2773 (14.1) | 2837 (14.4) |  |  |
| Current | 73944 (74.8) | 14826 (74.9) | 14641 (74.1) | 14963 (75.6) | 14832 (75.2) | 14682 (74.4) |  |  |
| Missing | 2880 (2.8) | 544 (2.7) | 581 (2.9) | 555 (2.7) | 608 (3) | 592 (2.9) |  |  |
| Family history of colorectal cancer |  |  |  |  |  |  | < 0.001 |  |
| No | 88113 (87.3) | 17541 (87) | 17623 (87.4) | 17577 (87.1) | 17674 (87.5) | 17698 (87.6) |  |  |
| Yes | 10300 (10.2) | 1963 (9.7) | 2045 (10.1) | 2132 (10.6) | 2082 (10.3) | 2078 (10.3) |  |  |
| Possibly | 2493 (2.5) | 657 (3.3) | 501 (2.5) | 461 (2.3) | 447 (2.2) | 427 (2.1) |  |  |
| Missing | 774 (0.8) | 175 (0.9) | 167 (0.8) | 166 (0.8) | 132 (0.6) | 134 (0.7) |  |  |
| Diabetes |  |  |  |  |  |  | 0.007 |  |
| No | 94353 (92.8) | 18861 (92.7) | 18884 (92.9) | 18944 (93.2) | 18892 (92.9) | 18772 (92.3) |  |  |
| Yes | 6801 (6.7) | 1355 (6.7) | 1334 (6.6) | 1305 (6.4) | 1336 (6.6) | 1471 (7.2) |  |  |
| Missing | 526 (0.5) | 120 (0.6) | 118 (0.6) | 87 (0.4) | 107 (0.5) | 94 (0.5) |  |  |
| History of colorectal polyps |  |  |  |  |  |  | 0.002 |  |
| No | 94305 (92.7) | 18738 (92.1) | 18824 (92.6) | 18902 (92.9) | 18927 (93.1) | 18914 (93) |  |  |
| Yes | 6762 (6.7) | 1463 (7.2) | 1379 (6.8) | 1333 (6.6) | 1276 (6.3) | 1311 (6.4) |  |  |
| Missing | 613 (0.6) | 135 (0.7) | 133 (0.7) | 101 (0.5) | 132 (0.6) | 112 (0.6) |  |  |
| History of colon comorbidities |  |  |  |  |  |  |  |  |
| No | 99439 (97.8) | 19849 (97.6) | 19871 (97.7) | 19897 (97.8) | 19914 (97.9) | 19908 (97.9) | 0.098 |  |
| Yes | 1355 (1.3) | 295 (1.5) | 268 (1.3) | 286 (1.4) | 257 (1.3) | 249 (1.2) |  |  |
| Missing | 886 (0.9) | 192 (0.9) | 197 (1) | 153 (0.8) | 164 (0.8) | 180 (0.9) |  |  |
| Total energy from diet (kcal/day) | 1607.0 (1222.0, 2101.0) | 1952.0 (1535.0, 2500.0) | 1375.0 (1029.0, 1800.0) | 1486.0 (1129.0, 1941.0) | 1542.0 (1178.0, 2001.0) | 1712.0 (1324.0, 2211.0) | < 0.001 |  |
| Red meat (g/day) | 47.8 (26.8, 80.2) | 65.4 (37.6, 105.7) | 41.4 (24.0, 68.6) | 44.3 (25.2, 72.7) | 44.1 (25.1, 71.9) | 47.7 (26.1, 81.9) | < 0.001 |  |
| Processed meat (g/day) | 10.8 (5.1, 22.5) | 15.7 (7.5, 30.8) | 9.4 (4.7, 19.1) | 9.9 (4.9, 20.3) | 9.7 (4.6, 20.2) | 10.5 (4.8, 22.6) | < 0.001 |  |
| Fruit (g/day) | 231.6 (128.8, 359.6) | 198.4 (102.0, 329.4) | 191.9 (103.5, 308.0) | 225.8 (130.3, 343.2) | 248.3 (147.0, 374.4) | 291.8 (174.1, 442.2) | < 0.001 |  |
| Vegetables (g/day) | 242.9 (159.1, 359.4) | 207.6 (132.8, 309.6) | 179.3 (112.8, 268.8) | 223.0 (153.2, 322.8) | 263.1 (185.7, 368.7) | 359.8 (259.8, 501.0) | < 0.001 |  |
| Whole grain (servings/day) | 1.0 (0.6, 1.6) | 1.1 (0.6, 1.7) | 0.9 (0.5, 1.4) | 1.0 (0.6, 1.5) | 1.0 (0.6, 1.5) | 1.1 (0.6, 1.6) | < 0.001 |  |
| Dairy (servings/day) | 1.1 (0.6, 1.8) | 1.3 (0.7, 2.2) | 0.9 (0.5, 1.6) | 1.0 (0.6, 1.7) | 1.0 (0.6, 1.7) | 1.1 (0.6, 1.8) | < 0.001 |  |
| Add sugars (tsp/day) | 10.2 (6.6, 15.6) | 13.9 (9.2, 21.1) | 9.2 (6.0, 13.8) | 9.4 (6.1, 14.1) | 9.2 (6.1, 13.9) | 9.9 (6.5, 15.0) | < 0.001 |  |
| Dietary fiber (g/day) | 16.5 (12.1, 22.2) | 17.2 (12.9, 22.9) | 13.6 (9.7, 18.5) | 15.5 (11.4, 20.6) | 16.8 (12.5, 22.1) | 19.9 (15.0, 26.4) | < 0.001 |  |
| Protein (% energy) | 15.3 (13.5, 17.2) | 15.0 (13.1, 16.9) | 15.2 (13.4, 17.1) | 15.4 (13.7, 17.3) | 15.5 (13.7, 17.3) | 15.5 (13.7, 17.4) | < 0.001 |  |
| Total fat (% energy) | 31.8 (26.6, 36.8) | 33.4 (28.1, 38.2) | 31.5 (26.4, 36.4) | 31.6 (26.6, 36.4) | 31.3 (26.3, 36.3) | 31.3 (26.0, 36.4) | < 0.001 |  |
| Carbohydrate (% energy) | 52.0 (45.9, 58.1) | 49.9 (43.8, 56.1) | 52.3 (46.2, 58.4) | 52.3 (46.5, 58.0) | 52.5 (46.5, 58.6) | 52.8 (46.6, 59.1) | < 0.001 |  |
| Glycemic load | 101.6 (76.7, 132.9) | 121.1 (95.0, 154.9) | 88.6 (66.0, 115.8) | 94.3 (71.7, 122.9) | 97.8 (74.0, 126.2) | 107.8 (82.4, 140.6) | < 0.001 |  |
| Glycemic index | 53.6 (51.5, 55.7) | 54.1 (51.9, 56.3) | 53.9 (51.7, 56.0) | 53.6 (51.6, 55.6) | 53.4 (51.3, 55.4) | 53.1 (51.1, 55.1) | < 0.001 |  |
| Calcium (mg/day) | 922.6 (600.9, 1337.0) | 962.3 (658.0, 1370.0) | 799.7 (504.6, 1215.0) | 897.1 (576.6, 1305.0) | 935.1 (607.3, 1343.0) | 1018.0 (674.8, 1437.0) | < 0.001 |  |
| Folate (mg/day) | 593.5 (351.8, 755.5) | 598.1 (361.1, 771.6) | 536.9 (293.2, 696.4) | 579.8 (332.8, 732.7) | 604.3 (362.7, 756.4) | 655.6 (414.0, 817.7) | < 0.001 |  |
| Magnesium (mg/day) | 354.0 (273.6, 446.4) | 386.6 (305.8, 485.0) | 311.6 (236.5, 394.5) | 336.3 (259.9, 422.7) | 351.2 (274.3, 437.6) | 388.1 (305.2, 485.5) | < 0.001 |  |
| Iron (mg/day) | 24.0 (13.6, 31.7) | 24.4 (14.8, 33.2) | 21.7 (11.4, 29.7) | 23.5 (12.8, 30.9) | 24.4 (13.7, 31.4) | 26.2 (15.1, 33.3) | < 0.001 |  |
| Vitamin D (mcg/day) | 10.8 (3.9, 13.5) | 10.6 (4.4, 14.1) | 10.0 (3.4, 13.1) | 10.8 (3.8, 13.3) | 11.0 (4.0, 13.4) | 11.2 (4.1, 13.7) | < 0.001 |  |
| Olive oil (g/day) | 0.0 (0.0, 0.5) | 0.0 (0.0, 0.2) | 0.0 (0.0, 0.3) | 0.0 (0.0, 0.5) | 0.0 (0.0, 0.6) | 0.0 (0.0, 0.8) | < 0.001 |  |
| Data are presented as median (IQR) or number (percentage). "Others" refers to Asian, Pacific Islander, or American Indian. DHQ, dietary history of questionnaire; BMI, body mass index. Energy from the diet was adjusted using the residual method. | | | | | | | |  |
|  |  |  |  |  |  |  |  |  |
|  |  |  |  |  |  |  |  |  |

**Supplementary Table S2. Baseline characteristics of study population according to quintiles of energy-adjusted tomato juice consumption in 101680 participants.**

|  |  | Quintiles of energy-adjusted tomato juice consumption (g/day) | | | | | |
| --- | --- | --- | --- | --- | --- | --- | --- |
| Variables | Overall | Q1 (≤-19.516) | Q2 (-19.516 to -14.884) | Q3 (-14.884 to -10.47) | Q4 (-10.469 to -2.969) | Q5 (≥-2.968) | *p* |
| Number of participants | 101680 | 20336 | 20336 | 20336 | 20336 | 20336 |  |
| Age at DHQ (years) | 65.0 (61.0, 70.0) | 65.0 (60.0, 69.0) | 65.0 (61.0, 70.0) | 65.0 (61.0, 70.0) | 65.0 (61.0, 70.0) | 65.0 (61.0, 70.0) | < 0.001 |
| Sex |  |  |  |  |  |  | < 0.001 |
| Male | 49441 (48.6) | 13246 (65.1) | 9514 (46.8) | 8333 (41) | 8071 (39.7) | 10277 (50.5) |  |
| Female | 52239 (51.4) | 7090 (34.9) | 10822 (53.2) | 12003 (59) | 12265 (60.3) | 10059 (49.5) |  |
| Trial arm |  |  |  |  |  |  | 0.043 |
| Intervention | 51767 (50.9) | 10540 (51.8) | 10339 (50.8) | 10311 (50.7) | 10240 (50.4) | 10337 (50.8) |  |
| Control | 49913 (49.1) | 9796 (48.2) | 9997 (49.2) | 10025 (49.3) | 10096 (49.6) | 9999 (49.2) |  |
| Race |  |  |  |  |  |  | < 0.001 |
| White, Non-Hispanic | 92465 (91.0) | 17879 (88) | 17990 (88.5) | 18639 (91.7) | 18983 (93.4) | 18974 (93.3) |  |
| Black, Non-Hispanic | 3352 (3.3) | 868 (4.3) | 772 (3.8) | 628 (3.1) | 517 (2.5) | 567 (2.8) |  |
| Hispanic | 1493 (1.5) | 351 (1.7) | 281 (1.4) | 270 (1.3) | 267 (1.3) | 324 (1.6) |  |
| Others | 4333 (4.3) | 1225 (6) | 1287 (6.3) | 792 (3.9) | 565 (2.8) | 464 (2.3) |  |
| Missing | 37 (0.0) | 13 (0.1) | 6 (0) | 7 (0) | 4 (0) | 7 (0) |  |
| Marital status |  |  |  |  |  |  | < 0.001 |
| Married | 79578 (78.3) | 16380 (80.5) | 16092 (79.1) | 15931 (78.3) | 15666 (77) | 15509 (76.3) |  |
| Unmarried | 21916 (21.6) | 3933 (19.3) | 4212 (20.7) | 4362 (21.4) | 4634 (22.8) | 4775 (23.5) |  |
| Missing | 186 (0.2) | 23 (0.1) | 32 (0.2) | 43 (0.2) | 36 (0.2) | 52 (0.3) |  |
| Education level |  |  |  |  |  |  | < 0.001 |
| ≤high school | 42909 (42.2) | 9201 (45.2) | 8768 (43.1) | 8455 (41.6) | 8185 (40.2) | 8300 (40.8) |  |
| ≥some college | 58574 (57.6) | 11107 (54.6) | 11538 (56.7) | 11832 (58.2) | 12113 (59.6) | 11984 (58.9) |  |
| Missing | 197 (0.2) | 28 (0.1) | 30 (0.1) | 49 (0.2) | 38 (0.2) | 52 (0.3) |  |
| BMI (kg/m2) |  |  |  |  |  |  | < 0.001 |
| <25 | 34426 (33.9) | 6303 (31) | 7331 (36) | 7394 (36.4) | 7053 (34.7) | 6345 (31.2) |  |
| ≥25 | 65915 (64.8) | 13756 (67.6) | 12745 (62.7) | 12699 (62.4) | 13025 (64) | 13690 (67.3) |  |
| Missing | 1339 (1.3) | 277 (1.4) | 260 (1.3) | 243 (1.2) | 258 (1.3) | 301 (1.5) |  |
| Aspirin use |  |  |  |  |  |  | < 0.001 |
| No | 53472 (52.6) | 10604 (52.1) | 10881 (53.5) | 10864 (53.4) | 10950 (53.8) | 10173 (50) |  |
| Yes | 47775 (47.0) | 9645 (47.4) | 9367 (46.1) | 9404 (46.2) | 9298 (45.7) | 10061 (49.5) |  |
| Missing | 433 (0.4) | 87 (0.4) | 88 (0.4) | 68 (0.3) | 88 (0.4) | 102 (0.5) |  |
| Cigarette smoking |  |  |  |  |  |  | < 0.001 |
| Never | 48532 (47.7) | 8776 (43.2) | 10022 (49.3) | 10133 (49.8) | 10134 (49.8) | 9467 (46.6) |  |
| Current | 9393 (9.2) | 2312 (11.4) | 1689 (8.3) | 1665 (8.2) | 1708 (8.4) | 2019 (9.9) |  |
| Former | 43742 (43.0) | 9244 (45.5) | 8622 (42.4) | 8534 (42) | 8493 (41.8) | 8849 (43.5) |  |
| Missing | 13 (0.0) | 4 (0) | 3 (0) | 4 (0) | 1 (0) | 1 (0) |  |
| Alcohol drinking |  |  |  |  |  |  | < 0.001 |
| Never | 10110 (10.2) | 1754 (8.9) | 2111 (10.7) | 2113 (10.7) | 2149 (10.8) | 1983 (10.1) |  |
| Former | 14746 (14.9) | 3357 (17) | 3166 (16.1) | 2923 (14.8) | 2661 (13.4) | 2639 (13.4) |  |
| Current | 73944 (74.8) | 14685 (74.2) | 14439 (73.2) | 14750 (74.5) | 15001 (75.7) | 15069 (76.5) |  |
| Missing | 2880 (2.8) | 540 (2.7) | 620 (3) | 550 (2.7) | 525 (2.6) | 645 (3.2) |  |
| Family history of colorectal cancer | |  |  |  |  |  | 0.022 |
| No | 88113 (87.3) | 17638 (87.4) | 17560 (87) | 17634 (87.4) | 17660 (87.5) | 17621 (87.4) |  |
| Yes | 10300 (10.2) | 1983 (9.8) | 2122 (10.5) | 2073 (10.3) | 2058 (10.2) | 2064 (10.2) |  |
| Possibly | 2493 (2.5) | 561 (2.8) | 512 (2.5) | 476 (2.4) | 459 (2.3) | 485 (2.4) |  |
| Missing | 774 (0.8) | 154 (0.8) | 142 (0.7) | 153 (0.8) | 159 (0.8) | 166 (0.8) |  |
| Diabetes |  |  |  |  |  |  | < 0.001 |
| No | 94353 (92.8) | 18809 (92.5) | 18994 (93.4) | 18975 (93.3) | 18919 (93) | 18656 (91.7) |  |
| Yes | 6801 (6.7) | 1424 (7) | 1245 (6.1) | 1258 (6.2) | 1315 (6.5) | 1559 (7.7) |  |
| Missing | 526 (0.5) | 103 (0.5) | 97 (0.5) | 103 (0.5) | 102 (0.5) | 121 (0.6) |  |
| History of colorectal polyps | |  |  |  |  |  | 0.236 |
| No | 94305 (92.7) | 18786 (92.4) | 18872 (92.8) | 18886 (92.9) | 18935 (93.1) | 18826 (92.6) |  |
| Yes | 6762 (6.7) | 1427 (7) | 1347 (6.6) | 1325 (6.5) | 1283 (6.3) | 1380 (6.8) |  |
| Missing | 613 (0.6) | 123 (0.6) | 117 (0.6) | 125 (0.6) | 118 (0.6) | 130 (0.6) |  |
| History of colon comorbidities | |  |  |  |  |  | 0.22 |
| No | 99439 (97.8) | 19887 (97.8) | 19897 (97.8) | 19872 (97.7) | 19924 (98) | 19859 (97.7) |  |
| Yes | 1355 (1.3) | 281 (1.4) | 267 (1.3) | 289 (1.4) | 239 (1.2) | 279 (1.4) |  |
| Missing | 886 (0.9) | 168 (0.8) | 172 (0.8) | 175 (0.9) | 173 (0.9) | 198 (1) |  |
| Total energy from diet (kcal/day) | 1607.0 (1222.0, 2101.0) | 2325.0 (1884.0, 2863.0) | 1538.0 (1272.0, 2069.0) | 1504.0 (1157.0, 1750.0) | 1234.0 (976.3, 1620.0) | 1535.0 (1187.0, 1998.0) | < 0.001 |
| Red meat (g/day) | 47.8 (26.8, 80.2) | 77.6 (46.2, 122.7) | 48.6 (27.8, 77.3) | 41.2 (23.9, 65.9) | 36.1 (21.4, 59.7) | 46.0 (25.8, 76.2) | < 0.001 |
| Processed meat (g/day) | 10.8 (5.1, 22.5) | 19.0 (8.9, 35.6) | 11.1 (5.2, 22.4) | 9.2 (4.6, 18.0) | 7.9 (4.1, 15.8) | 10.4 (5.0, 21.4) | < 0.001 |
| Fruit (g/day) | 231.6 (128.8, 359.6) | 265.9 (141.6, 420.2) | 236.2 (131.9, 360.2) | 214.3 (119.3, 333.7) | 201.7 (111.6, 315.7) | 244.6 (143.1, 374.2) | < 0.001 |
| Vegetables (g/day) | 242.9 (159.1, 359.4) | 288.9 (195.6, 419.6) | 225.8 (149.4, 327.8) | 210.6 (137.0, 305.3) | 199.3 (131.9, 294.2) | 307.5 (208.8, 447.8) | < 0.001 |
| Whole grain (servings/day) | 1.0 (0.6, 1.6) | 1.3 (0.8, 2.0) | 1.1 (0.6, 1.6) | 0.9 (0.5, 1.4) | 0.8 (0.5, 1.3) | 1.0 (0.5, 1.5) | < 0.001 |
| Dairy (servings/day) | 1.1 (0.6, 1.8) | 1.5 (0.9, 2.4) | 1.1 (0.6, 1.8) | 1.0 (0.6, 1.6) | 0.9 (0.5, 1.4) | 1.1 (0.6, 1.7) | < 0.001 |
| Add sugars (tsp/day) | 10.2 (6.6, 15.6) | 16.1 (10.9, 23.5) | 10.6 (7.2, 15.3) | 9.2 (6.1, 13.1) | 7.7 (5.2, 11.6) | 9.1 (6.0, 13.9) | < 0.001 |
| Dietary fiber (g/day) | 16.5 (12.1, 22.2) | 22.1 (17.1, 28.4) | 16.5 (12.5, 21.4) | 14.9 (11.0, 19.4) | 13.3 (9.7, 18.0) | 16.8 (12.3, 22.7) | < 0.001 |
| Protein (% energy) | 101.6 (76.7, 132.9) | 143.7 (118.2, 178.1) | 102.7 (82.7, 128.4) | 93.1 (71.3, 113.8) | 79.2 (61.5, 103.6) | 96.3 (73.3, 125.7) | < 0.001 |
| Total fat (% energy) | 15.3 (13.5, 17.2) | 15.0 (13.2, 16.9) | 15.2 (13.4, 17.1) | 15.3 (13.5, 17.2) | 15.4 (13.7, 17.4) | 15.5 (13.7, 17.4) | < 0.001 |
| Carbohydrate (% energy) | 31.8 (26.6, 36.8) | 33.9 (28.6, 38.8) | 32.1 (26.9, 37.0) | 31.4 (26.4, 36.4) | 31.0 (26.0, 35.9) | 30.8 (25.8, 35.7) | < 0.001 |
| Glycemic load | 52.0 (45.9, 58.1) | 49.9 (44.0, 55.8) | 52.2 (46.3, 58.2) | 52.6 (46.5, 58.5) | 52.7 (46.6, 58.9) | 52.4 (46.2, 58.7) | < 0.001 |
| Glycemic index | 53.6 (51.5, 55.7) | 54.1 (52.0, 56.3) | 54.0 (51.8, 56.0) | 53.7 (51.6, 55.8) | 53.4 (51.4, 55.4) | 52.9 (50.9, 54.9) | < 0.001 |
| Calcium (mg/day) | 922.6 (600.9, 1337.0) | 1105.0 (785.7, 1523.0) | 910.2 (601.6, 1332.0) | 865.1 (556.2, 1277.0) | 809.2 (499.9, 1223.0) | 904.4 (589.2, 1327.0) | < 0.001 |
| Folate (mg/day) | 593.5 (351.8, 755.5) | 681.4 (441.9, 859.6) | 597.2 (345.8, 747.2) | 562.2 (314.6, 716.9) | 547.7 (290.7, 686.5) | 607.0 (362.1, 769.6) | < 0.001 |
| Magnesium (mg/day) | 354.0 (273.6, 446.4) | 450.5 (367.9, 550.2) | 352.4 (281.1, 432.6) | 327.4 (253.1, 403.5) | 299.2 (231.3, 376.6) | 353.7 (274.9, 447.2) | < 0.001 |
| Iron (mg/day) | 24.0 (13.6, 31.7) | 27.5 (17.8, 36.1) | 23.9 (13.5, 31.6) | 22.4 (12.2, 30.4) | 22.2 (10.9, 29.0) | 24.6 (13.6, 31.7) | < 0.001 |
| Vitamin D (mcg/day) | 10.8 (3.9, 13.5) | 11.3 (4.9, 14.7) | 10.9 (3.8, 13.5) | 10.6 (3.5, 13.2) | 10.6 (3.3, 12.8) | 10.9 (4.0, 13.5) | < 0.001 |
| Olive oil (g/day) | 0.0 (0.0, 0.5) | 0.0 (0.0, 0.7) | 0.0 (0.0, 0.5) | 0.0 (0.0, 0.4) | 0.0 (0.0, 0.4) | 0.0 (0.0, 0.5) | < 0.001 |

Data are presented as median (IQR) or number (percentage). "Others" refers to Asian, Pacific Islander, or American Indian. DHQ, dietary history of questionnaire; BMI, body mass index.

Energy from the diet was adjusted using the residual method.

**Supplementary Table S3. Baseline characteristics of study population according to quintiles of energy-adjusted tomato catsup consumption in 101680 participants.**

|  |  | Quintiles of energy-adjusted tomato catsup consumption (g/day) | | | | | |
| --- | --- | --- | --- | --- | --- | --- | --- |
| Variables | Overall | Q1 (≤0.139) | Q2 (0.14 to 1.039) | Q3 (1.039 to 1.798) | Q4 (1.798 to 3.197) | Q5 (≥3.197) | *p* |
| Number of participants | 101680 | 20335 | 20337 | 20336 | 20336 | 20336 |  |
| Age at DHQ (years) | 65.0 (61.0, 70.0) | 65.0 (60.0, 70.0) | 65.0 (61.0, 70.0) | 66.0 (61.0, 70.0) | 65.0 (61.0, 70.0) | 64.0 (60.0, 69.0) | < 0.001 |
| Sex |  |  |  |  |  |  | < 0.001 |
| Male | 49441 (48.6) | 12535 (61.6) | 8788 (43.2) | 7384 (36.3) | 8232 (40.5) | 12502 (61.5) |  |
| Female | 52239 (51.4) | 7800 (38.4) | 11549 (56.8) | 12952 (63.7) | 12104 (59.5) | 7834 (38.5) |  |
| Trial arm |  |  |  |  |  |  | 0.084 |
| Intervention | 51767 (50.9) | 10438 (51.3) | 10246 (50.4) | 10305 (50.7) | 10294 (50.6) | 10484 (51.6) |  |
| Control | 49913 (49.1) | 9897 (48.7) | 10091 (49.6) | 10031 (49.3) | 10042 (49.4) | 9852 (48.4) |  |
| Race |  |  |  |  |  |  | < 0.001 |
| White, Non-Hispanic | 92465 (91.0) | 18325 (90.2) | 18588 (91.4) | 18406 (90.5) | 18305 (90.1) | 18841 (92.7) |  |
| Black, Non-Hispanic | 3352 (3.3) | 820 (4) | 668 (3.3) | 701 (3.4) | 701 (3.4) | 462 (2.3) |  |
| Hispanic | 1493 (1.5) | 411 (2) | 297 (1.5) | 313 (1.5) | 291 (1.4) | 181 (0.9) |  |
| Others | 4333 (4.3) | 771 (3.8) | 776 (3.8) | 910 (4.5) | 1030 (5.1) | 846 (4.2) |  |
| Missing | 37 (0.0) | 8 (0) | 8 (0) | 6 (0) | 9 (0) | 6 (0) |  |
| Marital status |  |  |  |  |  |  | < 0.001 |
| Married | 79578 (78.3) | 15817 (77.8) | 15736 (77.4) | 15520 (76.3) | 15717 (77.3) | 16788 (82.6) |  |
| Unmarried | 21916 (21.6) | 4483 (22) | 4571 (22.5) | 4776 (23.5) | 4578 (22.5) | 3508 (17.3) |  |
| Missing | 186 (0.2) | 35 (0.2) | 30 (0.1) | 40 (0.2) | 41 (0.2) | 40 (0.2) |  |
| Education level |  |  |  |  |  |  | < 0.001 |
| ≤high school | 42909 (42.2) | 7920 (38.9) | 8033 (39.5) | 8490 (41.7) | 9166 (45.1) | 9300 (45.7) |  |
| ≥some college | 58574 (57.6) | 12385 (60.9) | 12268 (60.3) | 11804 (58) | 11126 (54.7) | 10991 (54) |  |
| Missing | 197 (0.2) | 30 (0.1) | 36 (0.2) | 42 (0.2) | 44 (0.2) | 45 (0.2) |  |
| BMI (kg/m2) |  |  |  |  |  |  | < 0.001 |
| <25 | 34426 (33.9) | 6732 (33.1) | 7611 (37.4) | 7758 (38.1) | 6973 (34.3) | 5352 (26.3) |  |
| ≥25 | 65915 (64.8) | 13326 (65.5) | 12459 (61.3) | 12320 (60.6) | 13085 (64.3) | 14725 (72.4) |  |
| Missing | 1339 (1.3) | 277 (1.4) | 267 (1.3) | 258 (1.3) | 278 (1.4) | 259 (1.3) |  |
| Aspirin use |  |  |  |  |  |  | < 0.001 |
| No | 53472 (52.6) | 10388 (51.1) | 10758 (52.9) | 11071 (54.4) | 11026 (54.2) | 10229 (50.3) |  |
| Yes | 47775 (47.0) | 9851 (48.4) | 9496 (46.7) | 9180 (45.1) | 9225 (45.4) | 10023 (49.3) |  |
| Missing | 433 (0.4) | 96 (0.5) | 83 (0.4) | 85 (0.4) | 85 (0.4) | 84 (0.4) |  |
| Cigarette smoking |  |  |  |  |  |  | < 0.001 |
| Never | 48532 (47.7) | 8726 (42.9) | 9782 (48.1) | 10202 (50.2) | 10285 (50.6) | 9537 (46.9) |  |
| Current | 9393 (9.2) | 2236 (11) | 1783 (8.8) | 1734 (8.5) | 1713 (8.4) | 1927 (9.5) |  |
| Former | 43742 (43.0) | 9369 (46.1) | 8770 (43.1) | 8397 (41.3) | 8337 (41) | 8869 (43.6) |  |
| Missing | 13 (0.0) | 4 (0) | 2 (0) | 3 (0) | 1 (0) | 3 (0) |  |
| Alcohol drinking |  |  |  |  |  |  | < 0.001 |
| Never | 10110 (10.2) | 1589 (8) | 1949 (9.8) | 2226 (11.3) | 2364 (12) | 1982 (10) |  |
| Former | 14746 (14.9) | 2953 (14.9) | 2876 (14.5) | 2953 (15) | 2976 (15.1) | 2988 (15.1) |  |
| Current | 73944 (74.8) | 15297 (77.1) | 14980 (75.6) | 14548 (73.7) | 14329 (72.9) | 14790 (74.8) |  |
| Missing | 2880 (2.8) | 496 (2.4) | 532 (2.6) | 609 (3) | 667 (3.3) | 576 (2.8) |  |
| Family history of colorectal cancer | |  |  |  |  |  | < 0.001 |
| No | 88113 (87.3) | 17693 (87.7) | 17531 (86.8) | 17644 (87.4) | 17609 (87.3) | 17636 (87.4) |  |
| Yes | 10300 (10.2) | 1966 (9.7) | 2164 (10.7) | 2088 (10.3) | 2089 (10.4) | 1993 (9.9) |  |
| Possibly | 2493 (2.5) | 525 (2.6) | 494 (2.4) | 446 (2.2) | 476 (2.4) | 552 (2.7) |  |
| Missing | 774 (0.8) | 151 (0.7) | 148 (0.7) | 158 (0.8) | 162 (0.8) | 155 (0.8) |  |
| Diabetes |  |  |  |  |  |  | < 0.001 |
| No | 94353 (92.8) | 18932 (93.1) | 18976 (93.3) | 18973 (93.3) | 18781 (92.4) | 18691 (91.9) |  |
| Yes | 6801 (6.7) | 1280 (6.3) | 1243 (6.1) | 1258 (6.2) | 1469 (7.2) | 1551 (7.6) |  |
| Missing | 526 (0.5) | 123 (0.6) | 118 (0.6) | 105 (0.5) | 86 (0.4) | 94 (0.5) |  |
| History of colorectal polyps |  |  |  |  |  |  | 0.023 |
| No | 94305 (92.7) | 18815 (92.5) | 18808 (92.5) | 18874 (92.8) | 18982 (93.3) | 18826 (92.6) |  |
| Yes | 6762 (6.7) | 1385 (6.8) | 1395 (6.9) | 1339 (6.6) | 1248 (6.1) | 1395 (6.9) |  |
| Missing | 613 (0.6) | 135 (0.7) | 134 (0.7) | 123 (0.6) | 106 (0.5) | 115 (0.6) |  |
| History of colon comorbidities | |  |  |  |  |  | 0.962 |
| No | 99439 (97.8) | 19889 (97.8) | 19880 (97.8) | 19869 (97.7) | 19904 (97.9) | 19897 (97.8) |  |
| Yes | 1355 (1.3) | 269 (1.3) | 270 (1.3) | 287 (1.4) | 260 (1.3) | 269 (1.3) |  |
| Missing | 886 (0.9) | 177 (0.9) | 187 (0.9) | 180 (0.9) | 172 (0.8) | 170 (0.8) |  |
| Total energy from diet (kcal/day) | 1607.0 (1222.0, 2101.0) | 2323.0 (2001.0, 2791.0) | 1602.0 (1452.0, 1840.0) | 1236.0 (1100.0, 1535.0) | 1095.0 (827.7, 1493.0) | 1772.0 (1302.0, 2270.0) | < 0.001 |
| Red meat (g/day) | 47.8 (26.8, 80.2) | 72.2 (42.0, 113.5) | 46.0 (27.1, 71.8) | 36.2 (21.3, 57.6) | 33.5 (19.7, 56.8) | 62.9 (37.6, 99.8) | < 0.001 |
| Processed meat (g/day) | 10.8 (5.1, 22.5) | 17.1 (7.9, 32.9) | 10.3 (5.0, 20.8) | 8.1 (4.0, 16.2) | 7.5 (3.8, 15.0) | 14.6 (7.3, 27.7) | < 0.001 |
| Fruit (g/day) | 231.6 (128.8, 359.6) | 297.8 (163.0, 466.7) | 259.6 (151.1, 386.6) | 221.7 (129.0, 333.6) | 183.8 (100.6, 289.3) | 212.5 (117.1, 336.4) | < 0.001 |
| Vegetables (g/day) | 242.9 (159.1, 359.4) | 323.6 (221.4, 470.8) | 252.8 (173.1, 355.0) | 207.2 (140.7, 297.7) | 181.3 (115.7, 273.8) | 268.6 (181.6, 388.7) | < 0.001 |
| Whole grain (servings/day) | 1.0 (0.6, 1.6) | 1.4 (0.8, 2.0) | 1.1 (0.7, 1.6) | 0.9 (0.5, 1.3) | 0.7 (0.4, 1.2) | 1.0 (0.6, 1.6) | < 0.001 |
| Dairy (servings/day) | 1.1 (0.6, 1.8) | 1.6 (1.0, 2.5) | 1.2 (0.7, 1.8) | 0.9 (0.5, 1.5) | 0.7 (0.4, 1.3) | 1.1 (0.6, 1.8) | < 0.001 |
| Add sugars (tsp/day) | 10.2 (6.6, 15.6) | 15.3 (10.5, 22.1) | 10.5 (7.4, 14.8) | 8.2 (5.7, 11.8) | 7.1 (4.6, 11.1) | 11.3 (7.3, 17.0) | < 0.001 |
| Dietary fiber (g/day) | 16.5 (12.1, 22.2) | 23.2 (18.2, 29.5) | 17.3 (13.8, 21.6) | 14.1 (11.0, 18.0) | 12.1 (8.7, 16.6) | 17.2 (12.6, 22.8) | < 0.001 |
| Protein (% energy) | 15.3 (13.5, 17.2) | 15.1 (13.2, 17.0) | 15.3 (13.5, 17.2) | 15.3 (13.5, 17.3) | 15.4 (13.6, 17.2) | 15.4 (13.7, 17.2) | < 0.001 |
| Total fat (% energy) | 31.8 (26.6, 36.8) | 33.1 (27.6, 38.1) | 31.2 (26.1, 36.2) | 30.5 (25.4, 35.6) | 30.9 (25.8, 35.8) | 33.3 (28.6, 37.8) | < 0.001 |
| Carbohydrate (% energy) | 52.0 (45.9, 58.1) | 50.0 (43.7, 56.3) | 52.5 (46.5, 58.6) | 53.5 (47.5, 59.6) | 53.3 (47.2, 59.4) | 50.5 (45.0, 56.1) | < 0.001 |
| Glycemic load | 101.6 (76.7, 132.9) | 143.6 (120.7, 174.0) | 104.7 (89.6, 122.6) | 83.8 (69.9, 102.3) | 72.5 (54.5, 97.8) | 108.8 (81.4, 141.3) | < 0.001 |
| Glycemic index | 53.6 (51.5, 55.7) | 53.5 (51.3, 55.7) | 53.4 (51.3, 55.5) | 53.5 (51.4, 55.5) | 53.6 (51.5, 55.7) | 54.0 (52.0, 55.9) | < 0.001 |
| Calcium (mg/day) | 922.6 (600.9, 1337.0) | 1176.0 (840.4, 1601.0) | 972.2 (654.2, 1386.0) | 844.5 (534.9, 1254.0) | 737.8 (439.5, 1151.0) | 876.4 (588.9, 1276.0) | < 0.001 |
| Folate (mg/day) | 593.5 (351.8, 755.5) | 723.6 (474.7, 890.3) | 630.0 (366.4, 764.8) | 583.1 (307.3, 701.7) | 521.4 (263.6, 658.4) | 572.7 (344.6, 750.3) | < 0.001 |
| Magnesium (mg/day) | 354.0 (273.6, 446.4) | 472.7 (391.7, 565.9) | 366.6 (302.8, 432.8) | 312.6 (250.2, 376.3) | 277.4 (209.9, 353.7) | 361.5 (277.3, 455.0) | < 0.001 |
| Iron (mg/day) | 24.0 (13.6, 31.7) | 29.0 (18.5, 36.9) | 25.3 (14.0, 32.1) | 23.6 (11.6, 29.5) | 21.0 (9.9, 28.0) | 23.1 (13.7, 31.9) | < 0.001 |
| Vitamin D (mcg/day) | 10.8 (3.9, 13.5) | 12.0 (5.3, 15.2) | 11.3 (4.2, 13.8) | 10.9 (3.5, 13.0) | 9.7 (2.8, 12.4) | 9.6 (3.8, 13.4) | < 0.001 |
| Olive oil (g/day) | 0.0 (0.0, 0.5) | 0.0 (0.0, 1.2) | 0.0 (0.0, 0.7) | 0.0 (0.0, 0.4) | 0.0 (0.0, 0.3) | 0.0 (0.0, 0.3) | < 0.001 |

Data are presented as median (IQR) or number (percentage). "Others" refers to Asian, Pacific Islander, or American Indian. DHQ, dietary history of questionnaire; BMI, body mass index.

Energy from the diet was adjusted using the residual method.

**Supplementary Table S4. Baseline characteristics of study population according to quintiles of energy-adjusted lycopene intake in 101680 participants.**

|  |  | Quintiles of energy-adjusted lycopene intake (mcg/day) | | | | |
| --- | --- | --- | --- | --- | --- | --- |
| Variables | Overall | Q1 (≤3483.129) | Q2 (3483.133 to 4716.331) | Q3 (4716.353 to 5867.565) | Q4 (5867.603 to 7913.297) | Q5 (≥7913.508) |
| Number of participants | 101680 | 20336 | 20336 | 20336 | 20336 | 20336 |
| Age at DHQ (years) | 65.0 (61.0, 70.0) | 66.0 (61.0, 70.0) | 66.0 (61.0, 70.0) | 65.0 (61.0, 70.0) | 65.0 (60.0, 69.0) | 65.0 (60.0, 69.0) |
| Sex |  |  |  |  |  |  |
| Male | 49441 (48.6) | 12788 (62.9) | 9381 (46.1) | 8291 (40.8) | 8396 (41.3) | 10585 (52.1) |
| Female | 52239 (51.4) | 7548 (37.1) | 10955 (53.9) | 12045 (59.2) | 11940 (58.7) | 9751 (47.9) |
| Trial arm |  |  |  |  |  |  |
| Intervention | 51767 (50.9) | 10434 (51.3) | 10288 (50.6) | 10226 (50.3) | 10419 (51.2) | 10400 (51.1) |
| Control | 49913 (49.1) | 9902 (48.7) | 10048 (49.4) | 10110 (49.7) | 9917 (48.8) | 9936 (48.9) |
| Race |  |  |  |  |  |  |
| White, Non-Hispanic | 92465 (90.9) | 17815 (87.6) | 18272 (89.9) | 18748 (92.2) | 18969 (93.3) | 18661 (91.8) |
| Black, Non-Hispanic | 3352 (3.3) | 1163 (5.7) | 770 (3.8) | 513 (2.5) | 400 (2) | 506 (2.5) |
| Hispanic | 1493 (1.5) | 240 (1.2) | 220 (1.1) | 243 (1.2) | 300 (1.5) | 490 (2.4) |
| Others | 4333 (4.3) | 1112 (5.5) | 1067 (5.2) | 824 (4.1) | 663 (3.3) | 667 (3.3) |
| Missing | 37 (0.0) | 6 (0.0) | 7 (0.0) | 8 (0.0) | 4 (0.0) | 12 (0.1) |
| Marital status |  |  |  |  |  |  |
| Married | 79578 (78.3) | 15826 (77.8) | 15833 (77.9) | 15992 (78.6) | 16105 (79.2) | 15822 (77.8) |
| Unmarried | 21916 (21.6) | 4475 (22) | 4460 (21.9) | 4310 (21.2) | 4199 (20.6) | 4472 (22) |
| Missing | 186 (0.2) | 35 (0.2) | 43 (0.2) | 34 (0.2) | 32 (0.2) | 42 (0.2) |
| Education level |  |  |  |  |  |  |
| ≤high school | 42909 (42.2) | 9069 (44.6) | 8576 (42.2) | 8557 (42.1) | 8433 (41.5) | 8274 (40.7) |
| ≥some college | 58574 (57.6) | 11229 (55.2) | 11719 (57.6) | 11742 (57.7) | 11872 (58.4) | 12012 (59.1) |
| Missing | 197 (0.2) | 38 (0.2) | 41 (0.2) | 37 (0.2) | 31 (0.2) | 50 (0.2) |
| BMI (kg/m2) |  |  |  |  |  |  |
| <25 | 34426 (33.9) | 6944 (34.1) | 7593 (37.3) | 7156 (35.2) | 6713 (33) | 6020 (29.6) |
| ≥25 | 65915 (64.8) | 13104 (64.4) | 12502 (61.5) | 12929 (63.6) | 13344 (65.6) | 14036 (69) |
| Missing | 1339 (1.3) | 288 (1.4) | 241 (1.2) | 251 (1.2) | 279 (1.4) | 280 (1.4) |
| Aspirin use |  |  |  |  |  |  |
| No | 53472 (52.6) | 10700 (52.6) | 10861 (53.4) | 11015 (54.2) | 10722 (52.7) | 10174 (50) |
| Yes | 47775 (47.0) | 9528 (46.9) | 9384 (46.1) | 9249 (45.5) | 9544 (46.9) | 10070 (49.5) |
| Missing | 433 (0.4) | 108 (0.5) | 91 (0.4) | 72 (0.4) | 70 (0.3) | 92 (0.5) |
| Cigarette smoking |  |  |  |  |  |  |
| Never | 48532 (47.7) | 8711 (42.8) | 9987 (49.1) | 10249 (50.4) | 10130 (49.8) | 9455 (46.5) |
| Current | 9393 (9.2) | 2502 (12.3) | 1790 (8.8) | 1614 (7.9) | 1661 (8.2) | 1826 (9) |
| Former | 43742 (43.0) | 9117 (44.8) | 8558 (42.1) | 8470 (41.7) | 8543 (42) | 9054 (44.5) |
| Missing | 13 (0.0) | 6 (0) | 1 (0) | 3 (0) | 2 (0) | 1 (0) |
| Alcohol drinking |  |  |  |  |  |  |
| Never | 10110 (9.9) | 1929 (9.5) | 2083 (10.2) | 2146 (10.6) | 2021 (9.9) | 1931 (9.5) |
| Former | 14746 (14.5) | 3303 (16.2) | 3008 (14.8) | 2827 (13.9) | 2696 (13.3) | 2912 (14.3) |
| Current | 73944 (72.7) | 14554 (71.6) | 14692 (72.2) | 14786 (72.7) | 15045 (74) | 14867 (73.1) |
| Missing | 2880 (2.8) | 550 (2.7) | 553 (2.7) | 577 (2.8) | 574 (2.8) | 626 (3.1) |
| Family history of colorectal cancer | |  |  |  |  |  |
| No | 88113 (86.7) | 17553 (86.3) | 17566 (86.4) | 17700 (87) | 17639 (86.7) | 17655 (86.8) |
| Yes | 10300 (10.1) | 2005 (9.9) | 2073 (10.2) | 2050 (10.1) | 2133 (10.5) | 2039 (10) |
| Possibly | 2493 (2.5) | 608 (3) | 521 (2.6) | 428 (2.1) | 440 (2.2) | 496 (2.4) |
| Missing | 774 (0.8) | 170 (0.8) | 176 (0.9) | 158 (0.8) | 124 (0.6) | 146 (0.7) |
| Diabetes |  |  |  |  |  |  |
| No | 94353 (92.8) | 19007 (93.5) | 18986 (93.4) | 18943 (93.2) | 18820 (92.5) | 18597 (91.4) |
| Yes | 6801 (6.7) | 1220 (6) | 1235 (6.1) | 1295 (6.4) | 1424 (7) | 1627 (8) |
| Missing | 526 (0.5) | 109 (0.5) | 115 (0.6) | 98 (0.5) | 92 (0.5) | 112 (0.6) |
| History of colorectal polyps |  |  |  |  |  |  |
| No | 94305 (92.7) | 18753 (92.2) | 18808 (92.5) | 18913 (93) | 18956 (93.2) | 18875 (92.8) |
| Yes | 6762 (6.7) | 1456 (7.2) | 1392 (6.8) | 1310 (6.4) | 1269 (6.2) | 1335 (6.6) |
| Missing | 613 (0.6) | 127 (0.6) | 136 (0.7) | 113 (0.6) | 111 (0.5) | 126 (0.6) |
| History of colon comorbidities |  |  |  |  |  |  |
| No | 99439 (97.8) | 19873 (97.7) | 19883 (97.8) | 19916 (97.9) | 19890 (97.8) | 19877 (97.7) |
| Yes | 1355 (1.3) | 286 (1.4) | 271 (1.3) | 255 (1.3) | 275 (1.4) | 268 (1.3) |
| Missing | 886 (0.9) | 177 (0.9) | 182 (0.9) | 165 (0.8) | 171 (0.8) | 191 (0.9) |
| Total energy from diet (kcal/day) | 1607.0 (1222.0, 2101.0) | 2042.0 (1652.0, 2574.0) | 1495.0 (1185.0, 1893.0) | 1364.0 (1047.0, 1771.0) | 1435.0 (1083.0, 1876.0) | 1741.0 (1334.0, 2287.0) |
| Red meat (g/day) | 47.8 (26.8, 80.2) | 56.9 (31.9, 94.5) | 41.8 (23.9, 69.3) | 40.8 (23.9, 66.7) | 46.0 (26.5, 75.5) | 56.4 (30.6, 95.5) |
| Processed meat (g/day) | 10.8 (5.1, 22.5) | 15.5 (7.0, 31.2) | 9.9 (4.8, 20.6) | 9.1 (4.5, 18.3) | 9.5 (4.7, 19.3) | 11.7 (5.4, 23.9) |
| Fruit (g/day) | 231.6 (128.8, 359.6) | 226.9 (118.5, 363.6) | 217.4 (120.1, 334.9) | 208.4 (116.1, 326.4) | 223.6 (128.7, 345.0) | 282.4 (166.1, 439.4) |
| Vegetables (g/day) | 242.9 (159.1, 359.4) | 214.8 (140.7, 313.1) | 198.5 (130.5, 290.7) | 208.7 (139.3, 301.7) | 251.3 (173.9, 353.5) | 372.1 (264.9, 521.7) |
| Whole grain (servings/day) | 1.0 (0.6, 1.6) | 1.2 (0.7, 1.8) | 1.0 (0.6, 1.5) | 0.9 (0.5, 1.4) | 0.9 (0.5, 1.4) | 1.0 (0.6, 1.6) |
| Dairy (servings/day) | 1.1 (0.6, 1.8) | 1.4 (0.8, 2.5) | 1.1 (0.6, 1.8) | 0.9 (0.5, 1.5) | 0.9 (0.5, 1.5) | 1.1 (0.6, 1.7) |
| Add sugars (tsp/day) | 10.2 (6.6, 15.6) | 15.1 (9.9, 22.3) | 10.1 (6.8, 14.7) | 8.7 (5.7, 12.9) | 8.5 (5.6, 12.8) | 9.9 (6.6, 15.1) |
| Dietary fiber (g/day) | 16.5 (12.1, 22.2) | 18.2 (13.7, 23.8) | 15.1 (11.1, 19.9) | 14.3 (10.4, 19.2) | 15.8 (11.5, 21.1) | 20.0 (14.9, 26.9) |
| Protein (% energy) | 15.3 (13.5, 17.2) | 14.4 (12.4, 16.3) | 15.0 (13.3, 16.9) | 15.4 (13.7, 17.2) | 15.8 (14.1, 17.6) | 15.9 (14.1, 17.7) |
| Total fat (% energy) | 31.8 (26.6, 36.8) | 33.1 (27.4, 38.5) | 31.8 (26.5, 36.7) | 31.5 (26.5, 36.3) | 31.6 (26.7, 36.4) | 31.2 (26.2, 36.0) |
| Carbohydrate (% energy) | 52.0 (45.9, 58.1) | 50.5 (44.0, 56.8) | 52.4 (46.3, 58.5) | 52.4 (46.5, 58.3) | 52.0 (46.2, 58.0) | 52.4 (46.4, 58.7) |
| Glycemic load | 101.6 (76.7, 132.9) | 128.0 (102.1, 161.0) | 96.3 (75.6, 121.7) | 87.2 (66.1, 112.9) | 90.3 (68.0, 118.2) | 109.0 (82.7, 143.1) |
| Glycemic index | 53.6 (51.5, 55.7) | 54.2 (51.7, 56.5) | 53.9 (51.7, 56.0) | 53.7 (51.6, 55.7) | 53.4 (51.5, 55.3) | 53.0 (51.0, 54.9) |
| Calcium (mg/day) | 922.6 (600.9, 1337.0) | 1042.0 (713.3, 1470.0) | 892.6 (575.2, 1296.0) | 845.7 (534.6, 1260.0) | 859.9 (553.4, 1277.0) | 969.5 (645.6, 1383.0) |
| Folate (mg/day) | 593.5 (351.8, 755.5) | 621.8 (383.8, 792.6) | 572.3 (323.2, 724.7) | 560.3 (309.6, 706.3) | 575.8 (331.1, 731.8) | 652.8 (415.1, 825.2) |
| Magnesium (mg/day) | 354.0 (273.6, 446.4) | 404.5 (321.8, 501.8) | 335.9 (259.6, 416.3) | 316.5 (244.5, 397.0) | 330.3 (253.6, 415.3) | 391.5 (306.4, 493.7) |
| Iron (mg/day) | 24.0 (13.6, 31.7) | 25.0 (15.2, 33.7) | 23.0 (12.6, 30.8) | 22.7 (11.9, 30.0) | 23.2 (12.7, 30.6) | 26.2 (15.6, 33.9) |
| Vitamin D (mcg/day) | 10.8 (3.9, 13.5) | 11.3 (4.7, 14.6) | 10.8 (3.8, 13.5) | 10.7 (3.5, 13.1) | 10.5 (3.5, 13.0) | 10.9 (4.1, 13.6) |
| Olive oil (g/day) | 0.0 (0.0, 0.5) | 0.0 (0.0, 0.4) | 0.0 (0.0, 0.4) | 0.0 (0.0, 0.4) | 0.0 (0.0, 0.5) | 0.0 (0.0, 0.7) |

| **Supplementary Table S5. Subgroup analyses of the associations between energy-adjusted tomato-related products/lycopene intakes (Quintiles) and colorectal cancer incidence.** | | | | | | | | | | | | | | | | | | |  |  |
| --- | --- | --- | --- | --- | --- | --- | --- | --- | --- | --- | --- | --- | --- | --- | --- | --- | --- | --- | --- | --- |
|  |  |  |  |  |  |  |  |  |  |  |  |  |  |  |  |  |  |  |  | |
|  |  | Tomato juice (g/day) | |  | Raw tomato (g/day) |  | |  | Tomato salsa (g/day) | |  | Tomato catsup (g/day) | |  | Lycopene (mcg/day) |  |  |  | |  |
| Subgroups |  | HR (95%CI) | *p* | *P_interaction_* | HR (95%CI) | *p* | | *P_interaction_* | HR (95%CI) | *p* | *P_interaction_* | HR (95%CI) | *p* | *P_interaction_* | HR (95%CI) | *p* | *P_interaction_* |  | |  |
| Age, years |  |  |  | 0.055 |  |  | | 0.468 |  |  | 0.351 |  |  | 0.235 |  |  | 0.094 |  | |  |
| <60 | Q1 | 1(Ref) |  |  | 1(Ref) |  | |  | 1(Ref) |  |  | 1(Ref) |  |  | 1(Ref) |  |  |  | |  |
|  | Q2 | 1.1 (0.62~1.95) | 0.740 |  | 0.94 (0.59~1.51) | 0.798 | |  | 0.91 (0.53~1.55) | 0.721 |  | 0.63 (0.37~1.07) | 0.089 |  | 0.62 (0.34~1.15) | 0.130 |  |  | |  |
|  | Q3 | 1.61 (0.92~2.8) | 0.096 |  | 0.77 (0.46~1.27) | 0.303 | |  | 1.08 (0.62~1.88) | 0.777 |  | 0.83 (0.48~1.42) | 0.498 |  | 1.15 (0.69~1.94) | 0.591 |  |  | |  |
|  | Q4 | 2.31 (1.34~3.97) | 0.002 |  | 0.72 (0.43~1.21) | 0.215 |  | | 0.61 (0.34~1.1) | 0.101 |  | 0.97 (0.57~1.65) | 0.908 |  | 1.26 (0.77~2.08) | 0.354 |  |  | |  |
|  | Q5 | 1.51 (0.88~2.59) | 0.130 |  | 0.92 (0.58~1.47) | 0.738 | |  | 0.79 (0.47~1.3) | 0.351 |  | 0.52 (0.31~0.87) | 0.013 |  | 1.1 (0.67~1.79) | 0.706 |  |  | |  |
| >=60 | Q1 | 1(Ref) |  |  | 1(Ref) |  | |  | 1(Ref) |  |  | 1(Ref) |  |  | 1(Ref) |  |  |  | |  |
|  | Q2 | 0.95 (0.76~1.17) | 0.610 |  | 1.2 (0.97~1.48) | 0.092 | |  | 1.01 (0.82~1.26) | 0.898 |  | 0.9 (0.72~1.12) | 0.347 |  | 1.04 (0.85~1.28) | 0.683 |  |  | |  |
|  | Q3 | 0.97 (0.77~1.21) | 0.764 |  | 1.19 (0.96~1.46) | 0.108 | |  | 1.12 (0.89~1.41) | 0.325 |  | 1.05 (0.83~1.32) | 0.704 |  | 1.01 (0.82~1.25) | 0.912 |  |  | |  |
|  | Q4 | 0.88 (0.7~1.12) | 0.296 |  | 1.02 (0.83~1.27) | 0.824 | |  | 0.98 (0.78~1.23) | 0.833 |  | 1.02 (0.81~1.3) | 0.857 |  | 0.95 (0.77~1.18) | 0.672 |  |  | |  |
|  | Q5 | 0.96 (0.77~1.19) | 0.694 |  | 1.02 (0.82~1.26) | 0.865 | |  | 0.73 (0.57~0.92) | 0.008 |  | 0.91 (0.73~1.13) | 0.391 |  | 0.95 (0.78~1.17) | 0.643 |  |  | |  |
| Sex |  |  |  | 0.752 |  |  | | 0.804 |  |  | 0.703 |  |  | 0.527 |  |  | 0.303 |  | |  |
| Male | Q1 | 1(Ref) |  |  | 1(Ref) |  | |  | 1(Ref) |  |  | 1(Ref) |  |  | 1(Ref) |  |  |  | |  |
|  | Q2 | 1.03 (0.8~1.33) | 0.808 |  | 1.14 (0.9~1.46) | 0.279 | |  | 0.98 (0.76~1.27) | 0.893 |  | 0.88 (0.67~1.14) | 0.334 |  | 0.93 (0.72~1.2) | 0.578 |  |  | |  |
|  | Q3 | 1.05 (0.8~1.38) | 0.720 |  | 1.2 (0.94~1.52) | 0.147 | |  | 1.25 (0.96~1.64) | 0.095 |  | 1.09 (0.83~1.44) | 0.531 |  | 1.17 (0.91~1.5) | 0.225 |  |  | |  |
|  | Q4 | 1.01 (0.76~1.33) | 0.961 |  | 0.98 (0.76~1.27) | 0.869 | |  | 0.99 (0.76~1.3) | 0.950 |  | 0.94 (0.71~1.25) | 0.682 |  | 0.97 (0.74~1.25) | 0.792 |  |  | |  |
|  | Q5 | 1 (0.78~1.28) | 0.974 |  | 1.07 (0.84~1.37) | 0.598 | |  | 0.8 (0.61~1.04) | 0.093 |  | 0.81 (0.64~1.03) | 0.090 |  | 1.02 (0.81~1.3) | 0.839 |  |  | |  |
| Female | Q1 | 1(Ref) |  |  | 1(Ref) |  | |  | 1(Ref) |  |  | 1(Ref) |  |  | 1(Ref) |  |  |  | |  |
|  | Q2 | 0.89 (0.63~1.27) | 0.527 |  | 1.11 (0.81~1.53) | 0.521 | |  | 0.92 (0.66~1.29) | 0.637 |  | 0.83 (0.59~1.16) | 0.282 |  | 1.04 (0.77~1.42) | 0.796 |  |  | |  |
|  | Q3 | 1.01 (0.71~1.43) | 0.974 |  | 0.99 (0.72~1.36) | 0.931 | |  | 0.85 (0.59~1.24) | 0.402 |  | 0.92 (0.64~1.33) | 0.667 |  | 0.88 (0.64~1.21) | 0.423 |  |  | |  |
|  | Q4 | 1.01 (0.7~1.45) | 0.970 |  | 0.92 (0.67~1.27) | 0.624 | |  | 0.72 (0.5~1.05) | 0.086 |  | 1.03 (0.7~1.52) | 0.885 |  | 0.99 (0.73~1.35) | 0.958 |  |  | |  |
|  | Q5 | 1.04 (0.73~1.48) | 0.813 |  | 0.9 (0.65~1.24) | 0.511 | |  | 0.6 (0.41~0.87) | 0.007 |  | 0.87 (0.6~1.26) | 0.466 |  | 0.9 (0.65~1.24) | 0.505 |  |  | |  |
| Cigarette smoking |  |  |  | 0.212 |  |  | | 0.237 |  |  | 0.712 |  |  | 0.953 |  |  | 0.038 |  | |  |
| Never | Q1 | 1(Ref) |  |  | 1(Ref) |  | |  | 1(Ref) |  |  | 1(Ref) |  |  | 1(Ref) |  |  |  | |  |
|  | Q2 | 0.9 (0.66~1.23) | 0.517 |  | 1.27 (0.95~1.69) | 0.112 | |  | 0.95 (0.71~1.29) | 0.758 |  | 0.85 (0.62~1.18) | 0.334 |  | 1.09 (0.82~1.44) | 0.572 |  |  | |  |
|  | Q3 | 0.91 (0.66~1.26) | 0.568 |  | 1.04 (0.77~1.41) | 0.794 | |  | 1.04 (0.75~1.43) | 0.832 |  | 0.96 (0.69~1.34) | 0.815 |  | 0.83 (0.61~1.13) | 0.239 |  |  | |  |
|  | Q4 | 0.88 (0.63~1.22) | 0.441 |  | 1.06 (0.79~1.43) | 0.698 | |  | 0.8 (0.57~1.11) | 0.178 |  | 1.04 (0.74~1.46) | 0.830 |  | 1.02 (0.77~1.37) | 0.869 |  |  | |  |
|  | Q5 | 0.96 (0.7~1.31) | 0.805 |  | 0.96 (0.71~1.31) | 0.819 | |  | 0.64 (0.45~0.9) | 0.010 |  | 0.91 (0.67~1.24) | 0.550 |  | 0.82 (0.6~1.11) | 0.191 |  |  | |  |
| Current | Q1 | 1(Ref) |  |  | 1(Ref) |  | |  | 1(Ref) |  |  | 1(Ref) |  |  | 1(Ref) |  |  |  | |  |
|  | Q2 | 0.63 (0.31~1.28) | 0.202 |  | 1.01 (0.59~1.73) | 0.967 | |  | 1.1 (0.58~2.08) | 0.773 |  | 0.75 (0.41~1.38) | 0.350 |  | 0.57 (0.3~1.08) | 0.083 |  |  | |  |
|  | Q3 | 1.56 (0.86~2.84) | 0.144 |  | 0.82 (0.46~1.46) | 0.498 | |  | 1.39 (0.72~2.7) | 0.327 |  | 0.66 (0.34~1.28) | 0.219 |  | 0.92 (0.52~1.63) | 0.770 |  |  | |  |
|  | Q4 | 0.91 (0.46~1.78) | 0.775 |  | 0.73 (0.4~1.35) | 0.316 | |  | 1.07 (0.53~2.16) | 0.849 |  | 0.69 (0.35~1.37) | 0.294 |  | 0.56 (0.29~1.09) | 0.088 |  |  | |  |
|  | Q5 | 0.77 (0.41~1.45) | 0.417 |  | 0.58 (0.3~1.13) | 0.107 | |  | 1.32 (0.69~2.52) | 0.393 |  | 0.58 (0.31~1.09) | 0.088 |  | 0.99 (0.58~1.68) | 0.974 |  |  | |  |
| Former | Q1 | 1(Ref) |  |  | 1(Ref) |  | |  | 1(Ref) |  |  | 1(Ref) |  |  | 1(Ref) |  |  |  | |  |
|  | Q2 | 1.11 (0.83~1.49) | 0.496 |  | 1.07 (0.8~1.43) | 0.641 | |  | 0.99 (0.74~1.32) | 0.934 |  | 0.88 (0.66~1.19) | 0.420 |  | 1.01 (0.75~1.35) | 0.967 |  |  | |  |
|  | Q3 | 1.05 (0.77~1.44) | 0.750 |  | 1.28 (0.97~1.69) | 0.077 | |  | 1.1 (0.81~1.49) | 0.553 |  | 1.15 (0.85~1.57) | 0.358 |  | 1.31 (0.98~1.74) | 0.064 |  |  | |  |
|  | Q4 | 1.22 (0.89~1.66) | 0.221 |  | 0.96 (0.72~1.29) | 0.787 | |  | 0.98 (0.73~1.33) | 0.914 |  | 1.06 (0.78~1.46) | 0.701 |  | 1.1 (0.82~1.48) | 0.517 |  |  | |  |
|  | Q5 | 1.15 (0.86~1.53) | 0.351 |  | 1.15 (0.87~1.51) | 0.324 | |  | 0.75 (0.55~1.01) | 0.056 |  | 0.84 (0.63~1.13) | 0.252 |  | 1.14 (0.87~1.5) | 0.346 |  |  | |  |
| BMI, kg/m2 |  |  |  | 0.990 |  |  | | 0.468 |  |  | 0.957 |  |  | 0.186 |  |  | 0.580 |  | |  |
| <25 | Q1 | 1(Ref) |  |  | 1(Ref) |  | |  | 1(Ref) |  |  | 1(Ref) |  |  | 1(Ref) |  |  |  | |  |
|  | Q2 | 0.85 (0.59~1.23) | 0.386 |  | 1.27 (0.9~1.8) | 0.174 | |  | 0.94 (0.65~1.35) | 0.723 |  | 0.63 (0.44~0.91) | 0.013 |  | 0.91 (0.65~1.27) | 0.571 |  |  | |  |
|  | Q3 | 0.88 (0.6~1.29) | 0.514 |  | 1.13 (0.8~1.61) | 0.482 | |  | 0.99 (0.66~1.48) | 0.949 |  | 0.58 (0.39~0.87) | 0.008 |  | 0.95 (0.67~1.34) | 0.764 |  |  | |  |
|  | Q4 | 0.81 (0.54~1.22) | 0.312 |  | 0.86 (0.59~1.25) | 0.427 | |  | 0.79 (0.53~1.19) | 0.264 |  | 0.74 (0.49~1.11) | 0.151 |  | 0.76 (0.53~1.1) | 0.145 |  |  | |  |
|  | Q5 | 0.87 (0.59~1.27) | 0.469 |  | 0.97 (0.67~1.39) | 0.849 | |  | 0.75 (0.5~1.12) | 0.160 |  | 0.61 (0.41~0.91) | 0.014 |  | 0.88 (0.62~1.25) | 0.475 |  |  | |  |
| >=25 | Q1 | 1(Ref) |  |  | 1(Ref) |  | |  | 1(Ref) |  |  | 1(Ref) |  |  | 1(Ref) |  |  |  | |  |
|  | Q2 | 1 (0.78~1.28) | 0.987 |  | 1.1 (0.87~1.39) | 0.434 | |  | 0.99 (0.78~1.26) | 0.960 |  | 0.95 (0.74~1.22) | 0.692 |  | 1.04 (0.82~1.32) | 0.744 |  |  | |  |
|  | Q3 | 1.12 (0.87~1.44) | 0.381 |  | 1.12 (0.88~1.41) | 0.355 | |  | 1.15 (0.89~1.48) | 0.280 |  | 1.24 (0.96~1.61) | 0.094 |  | 1.09 (0.85~1.39) | 0.490 |  |  | |  |
|  | Q4 | 1.11 (0.86~1.44) | 0.424 |  | 1.04 (0.83~1.32) | 0.714 | |  | 0.96 (0.75~1.24) | 0.771 |  | 1.11 (0.85~1.44) | 0.448 |  | 1.14 (0.9~1.44) | 0.278 |  |  | |  |
|  | Q5 | 1.08 (0.85~1.37) | 0.526 |  | 1.02 (0.81~1.29) | 0.839 | |  | 0.74 (0.57~0.95) | 0.020 |  | 0.93 (0.74~1.18) | 0.564 |  | 1.04 (0.83~1.31) | 0.735 |  |  | |  |
| HR, hazard ratio; CI, confidence interval; BMI, body mass index. Adjusted for age (continuous), sex (male vs. female), trial arm (intervention vs. control), race (white, non-Hispanic vs. black, non-Hispanic vs. Hispanic vs. others), marital status (married vs. unmarried), education level (≤high school vs. ≥some college), aspirin use (no vs. yes), diabetes (no vs. yes), cigarette smoking (never vs. current vs. former), BMI (<25kg/m2 vs. ≥25kg/m2), family history of colorectal cancer (yes vs. no vs. possibly), alcohol drinking (never vs. former vs. current), history of colorectal polyps (no vs. yes), history of colon comorbidities (no vs. yes), and energy from diet (continuous). In subgroup analyses stratified by age, sex, trial arm, aspirin use, cigarette smoking, BMI, alcohol drinking, family history of colorectal cancer, history of colorectal polyps, history of colon comorbidities, and diabetes, HRs were adjusted except for the stratification factor. *P*-value for interaction was calculated by the like-hood ratio test, and *P*>0.05 was considered no statistical significance. | | | | | | | | | | | | | | | | | | |  | |
|  |  |  |  |  |  |  |  |  |  |  |  |  |  |  |  |  |  |  |  | |
|  |  |  |  |  |  |  |  |  |  |  |  |  |  |  |  |  |  |  |  | |
|  |  |  |  |  |  |  |  |  |  |  |  |  |  |  |  |  |  |  |  | |
|  |  |  |  |  |  |  |  |  |  |  |  |  |  |  |  |  |  |  |  | |
|  |  |  |  |  |  |  |  |  |  |  |  |  |  |  |  |  |  |  |  | |
|  |  |  |  |  |  |  |  |  |  |  |  |  |  |  |  |  |  |  |  | |
|  |  |  |  |  |  |  | |  |  |  |  |  |  |  |  |  |  |  | |  |

**Supplementary Table S6. Sensitivity analyses on the association between energy-adjusted tomato-related products/lycopene intakes and colorectal cancer incidence.**

|  | Primary analysis | Adjusted hazard ratio (95% confidence interval) ^a^ | | | | | |
| --- | --- | --- | --- | --- | --- | --- | --- |
|  |  | Excluding events within the first 2 years of follow-up (excluded 1636 participants) | Excluding events with extreme values of energy intake (excluded 2886 participants) | Additional Model 1 | Additional Model 2 | Additional Model 3 | Additional Model 4 |
| Raw tomato (g/day) |  |  |  |  |  |  |  |
| Q1 | 1(Ref) | 1(Ref) | 1(Ref) | 1(Ref) | 1(Ref) | 1(Ref) | 1(Ref) |
| Q2 | 1.14 (0.94~1.37) | 1.13 (0.92~1.4) | 1.09 (0.9~1.32) | 1.14 (0.94~1.39) | 1.14 (0.94~1.38) | 1.14 (0.94~1.38) | 1.14 (0.94~1.38) |
| Q3 | 1.1 (0.91~1.33) | 1.06 (0.86~1.32) | 1.05 (0.86~1.27) | 1.11 (0.92~1.35) | 1.11 (0.92~1.35) | 1.11 (0.92~1.35) | 1.12 (0.92~1.36) |
| Q4 | 0.96 (0.79~1.16) | 0.87 (0.69~1.09) | 0.92 (0.75~1.12) | 0.98 (0.8~1.19) | 0.98 (0.8~1.19) | 0.98 (0.8~1.2) | 0.98 (0.8~1.2) |
| Q5 | 0.99 (0.81~1.2) | 0.92 (0.74~1.15) | 0.97 (0.8~1.18) | 1.03 (0.83~1.26) | 1.03 (0.83~1.26) | 1.03 (0.83~1.27) | 1.03 (0.83~1.27) |
| *P* for trend | 0.355 | 0.083 | 0.272 | 0.624 | 0.631 | 0.646 | 0.664 |
| continuous (log2) | 0.98 (0.94~1.02) | 0.97 (0.93~1.01) | 0.98 (0.94~1.02) | 0.99 (0.94~1.03) | 0.99 (0.94~1.03) | 0.99 (0.94~1.03) | 0.99 (0.94~1.03) |
| Tomato salsa (g/day) | |  |  |  |  |  |  |
| Q1 | 1(Ref) | 1(Ref) | 1(Ref) | 1(Ref) | 1(Ref) | 1(Ref) | 1(Ref) |
| Q2 | 1.01 (0.83~1.23) | 1.02 (0.82~1.27) | 0.98 (0.8~1.2) | 1.01 (0.83~1.23) | 1.01 (0.82~1.23) | 1.01 (0.83~1.23) | 1.01 (0.83~1.23) |
| Q3 | 1.15 (0.93~1.43) | 1.13 (0.89~1.43) | 1.09 (0.88~1.36) | 1.14 (0.92~1.41) | 1.12 (0.91~1.39) | 1.13 (0.91~1.4) | 1.13 (0.91~1.4) |
| Q4 | 0.97 (0.79~1.2) | 0.92 (0.72~1.17) | 0.89 (0.72~1.11) | 0.97 (0.78~1.2) | 0.96 (0.77~1.19) | 0.96 (0.78~1.19) | 0.96 (0.78~1.19) |
| Q5 | 0.8 (0.65~0.99) | 0.78 (0.61~0.99) | 0.74 (0.6~0.93) | 0.8 (0.64~0.99) | 0.79 (0.64~0.98) | 0.8 (0.64~0.99) | 0.8 (0.64~0.99) |
| *P* for trend | 0.028 | 0.019 | 0.003 | 0.027 | 0.024 | 0.03 | 0.03 |
| continuous (log2) | 0.97 (0.94~1.01) | 0.96 (0.93~1) | 0.97 (0.94~1) | 0.97 (0.94~1.01) | 0.97 (0.94~1.01) | 0.97 (0.94~1.01) | 0.97 (0.94~1.01) |
| Tomato juice (g/day) |  |  |  |  |  |  |  |
| Q1 | 1(Ref) | 1(Ref) | 1(Ref) | 1(Ref) | 1(Ref) | 1(Ref) | 1(Ref) |
| Q2 | 0.97 (0.8~1.19) | 1 (0.8~1.26) | 1.02 (0.83~1.25) | 0.99 (0.81~1.21) | 0.98 (0.8~1.21) | 0.98 (0.8~1.21) | 0.98 (0.8~1.21) |
| Q3 | 1.05 (0.85~1.29) | 1.15 (0.91~1.45) | 1.07 (0.86~1.32) | 1.06 (0.86~1.31) | 1.06 (0.85~1.3) | 1.06 (0.86~1.31) | 1.06 (0.86~1.3) |
| Q4 | 1.04 (0.84~1.29) | 1.07 (0.83~1.36) | 1.03 (0.82~1.28) | 1.05 (0.85~1.31) | 1.04 (0.84~1.29) | 1.04 (0.84~1.3) | 1.04 (0.84~1.29) |
| Q5 | 1.03 (0.84~1.26) | 1.01 (0.8~1.27) | 1.04 (0.85~1.28) | 1.08 (0.87~1.33) | 1.06 (0.86~1.31) | 1.06 (0.86~1.31) | 1.06 (0.86~1.31) |
| *P* for trend | 0.612 | 0.895 | 0.733 | 0.384 | 0.455 | 0.469 | 0.48 |
| continuous (log2) | 1.02 (0.96~1.08) | 1.02 (0.95~1.09) | 1.02 (0.96~1.08) | 1.03 (0.96~1.1) | 1.03 (0.97~1.1) | 1.03 (0.96~1.1) | 1.03 (0.96~1.1) |
| Tomato catsup (g/day) | |  |  |  |  |  |  |
| Q1 | 1(Ref) | 1(Ref) | 1(Ref) | 1(Ref) | 1(Ref) | 1(Ref) | 1(Ref) |
| Q2 | 0.86 (0.7~1.05) | 0.91 (0.72~1.14) | 0.84 (0.68~1.04) | 0.85 (0.69~1.05) | 0.85 (0.69~1.04) | 0.85 (0.69~1.05) | 0.85 (0.69~1.04) |
| Q3 | 1.02 (0.83~1.26) | 1.13 (0.89~1.43) | 0.97 (0.78~1.21) | 1 (0.81~1.24) | 0.99 (0.8~1.23) | 0.99 (0.8~1.23) | 0.99 (0.8~1.23) |
| Q4 | 1.03 (0.83~1.28) | 1.04 (0.81~1.34) | 0.99 (0.79~1.25) | 1 (0.8~1.24) | 0.99 (0.79~1.23) | 0.98 (0.79~1.23) | 0.98 (0.78~1.22) |
| Q5 | 0.86 (0.7~1.05) | 0.95 (0.76~1.19) | 0.84 (0.69~1.04) | 0.84 (0.68~1.02) | 0.84 (0.68~1.03) | 0.84 (0.68~1.02) | 0.83 (0.68~1.02) |
| *P* for trend | 0.5 | 0.997 | 0.415 | 0.317 | 0.309 | 0.289 | 0.265 |
| continuous (log2) | 0.99 (0.95~1.03) | 1 (0.95~1.04) | 0.99 (0.95~1.03) | 0.98 (0.94~1.02) | 0.98 (0.94~1.02) | 0.98 (0.94~1.02) | 0.98 (0.94~1.02) |
| Lycopene (mcg/day) |  |  |  |  |  |  |  |
| Q1 | 1(Ref) | 1(Ref) | 1(Ref) | 1(Ref) | 1(Ref) | 1(Ref) | 1(Ref) |
| Q2 | 1.01 (0.83~1.22) | 1 (0.8~1.26) | 0.98 (0.8~1.19) | 1.01 (0.83~1.22) | 1 (0.83~1.22) | 1 (0.82~1.22) | 1 (0.82~1.22) |
| Q3 | 1.06 (0.87~1.29) | 1.15 (0.91~1.45) | 1.02 (0.83~1.24) | 1.05 (0.86~1.29) | 1.05 (0.86~1.29) | 1.05 (0.86~1.29) | 1.05 (0.86~1.28) |
| Q4 | 1.04 (0.86~1.27) | 1.07 (0.83~1.36) | 1.03 (0.85~1.26) | 1.03 (0.84~1.27) | 1.03 (0.84~1.27) | 1.03 (0.84~1.27) | 1.03 (0.84~1.27) |
| Q5 | 1.01 (0.84~1.22) | 1.01 (0.8~1.27) | 0.98 (0.81~1.19) | 1.02 (0.82~1.27) | 1.02 (0.82~1.27) | 1.01 (0.82~1.26) | 1.01 (0.81~1.26) |
| *P* for trend | 0.818 | 0.895 | 0.924 | 0.767 | 0.77 | 0.826 | 0.835 |
| continuous (log2) | 0.97 (0.92~1.03) | 0.97 (0.91~1.04) | 0.97 (0.91~1.03) | 0.98 (0.91~1.05) | 0.97 (0.91~1.05) | 0.97 (0.91~1.04) | 0.97 (0.91~1.04) |

Extreme values of energy intake were included <800 or >4000 kcal/day for males and <500 or >3500 kcal/day for females.

Additional Model 1: adjusted for the factors listed in the fully-adjusted model (table 2, model 3), and red meat (g/day), processed meat (g/day), fruit (g/day), vegetables (g/day), whole grain (servings/day), dairy (servings/day), sugar (tsp/day), and dietary fiber (g/day).

Additional Model 2: adjusted for additional Model 1 plus glycemic load, glycemic index, protein (% energy), total fat (% energy), and carbohydrate (% energy).

Additional Model 3: adjusted for additional Model 2 plus total calcium (mg/day), folate (mg/day), magnesium (mg/day), iron (mg/day), and vitamin D (mcg/day).

Additional Model 4: adjusted additional Model 3, and further adjusted olive oil (g/day). All covariates above-mentioned were treated as continuous variables in the models.

| Supplementary Table S7. Subgroup analyses of the associations between quintiles of energy-adjusted tomato-related products/ dietary lycopene intakes (Quintiles) and colorectal cancer mortality. | | | | | | | | | | | | | | | | | |  |  |
| --- | --- | --- | --- | --- | --- | --- | --- | --- | --- | --- | --- | --- | --- | --- | --- | --- | --- | --- | --- |
|  |  |  |  |  |  |  |  |  |  |  |  |  |  |  |  |  |  |  | |
|  |  | Tomato juice (g/day) |  |  | Raw tomato (g/day) |  |  | Tomato salsa (g/day) |  |  | Tomato catsup (g/day) |  |  | Lycopene (mcg/day) |  |  |  | |  |
| Subgroup |  | HR (95%CI) | *p* | *P_interaction_* | HR (95%CI) | *p* | *P_interaction_* | HR (95%CI) | *p* | *P_interaction_* | HR (95%CI) | *p* | *P_interaction_* | HR (95%CI) | *p* | *P_interaction_* |  | |  |
| Age, years |  |  |  | 0.630 |  |  | 0.285 |  |  | 0.814 |  |  | 0.532 |  |  | 0.618 |  | |  |
| <60 | Q1 | 1(Ref) |  |  | 1(Ref) |  |  | 1(Ref) |  |  | 1(Ref) |  |  | 1(Ref) |  |  |  | |  |
|  | Q2 | 1.01 (0.42~2.42) | 0.989 |  | 1.58 (0.7~3.57) | 0.270 |  | 0.89 (0.35~2.25) | 0.808 |  | 0.34 (0.13~0.87) | 0.024 |  | 0.85 (0.34~2.14) | 0.726 |  |  | |  |
|  | Q3 | 1.05 (0.42~2.62) | 0.914 |  | 1.01 (0.41~2.51) | 0.976 |  | 0.95 (0.37~2.47) | 0.923 |  | 0.45 (0.18~1.13) | 0.090 |  | 0.76 (0.29~1.94) | 0.559 |  |  | |  |
|  | Q4 | 1.38 (0.56~3.39) | 0.480 |  | 1.23 (0.51~2.97) | 0.644 |  | 0.6 (0.22~1.61) | 0.312 |  | 0.59 (0.24~1.43) | 0.240 |  | 1.02 (0.43~2.41) | 0.972 |  |  | |  |
|  | Q5 | 1.01 (0.41~2.49) | 0.981 |  | 1.63 (0.73~3.66) | 0.231 |  | 1.01 (0.44~2.33) | 0.984 |  | 0.4 (0.17~0.93) | 0.033 |  | 1.49 (0.68~3.27) | 0.320 |  |  | |  |
| >=60 | Q1 | 1(Ref) |  |  | 1(Ref) |  |  | 1(Ref) |  |  | 1(Ref) |  |  | 1(Ref) |  |  |  | |  |
|  | Q2 | 1.05 (0.75~1.47) | 0.766 |  | 1.14 (0.82~1.59) | 0.422 |  | 0.78 (0.56~1.1) | 0.153 |  | 1.04 (0.74~1.48) | 0.807 |  | 1.3 (0.95~1.78) | 0.100 |  |  | |  |
|  | Q3 | 1.08 (0.76~1.54) | 0.656 |  | 1.33 (0.97~1.83) | 0.074 |  | 1.06 (0.75~1.49) | 0.760 |  | 1.38 (0.97~1.97) | 0.077 |  | 1 (0.71~1.41) | 1.000 |  |  | |  |
|  | Q4 | 0.95 (0.66~1.38) | 0.788 |  | 1.16 (0.84~1.6) | 0.374 |  | 0.81 (0.57~1.16) | 0.246 |  | 1.44 (1~2.06) | 0.050 |  | 0.91 (0.64~1.29) | 0.591 |  |  | |  |
|  | Q5 | 1.08 (0.77~1.51) | 0.654 |  | 0.85 (0.61~1.2) | 0.368 |  | 0.86 (0.62~1.21) | 0.388 |  | 1.02 (0.73~1.43) | 0.917 |  | 1.16 (0.85~1.58) | 0.359 |  |  | |  |
| Sex |  |  |  | 0.646 |  |  | 0.725 |  |  | 0.713 |  |  | 0.749 |  |  | 0.205 |  | |  |
| Male | Q1 | 1(Ref) |  |  | 1(Ref) |  |  | 1(Ref) |  |  | 1(Ref) |  |  | 1(Ref) |  |  |  | |  |
|  | Q2 | 1.18 (0.8~1.73) | 0.398 |  | 1.12 (0.77~1.63) | 0.543 |  | 0.8 (0.53~1.21) | 0.287 |  | 0.92 (0.61~1.4) | 0.711 |  | 1.02 (0.7~1.5) | 0.900 |  |  | |  |
|  | Q3 | 1.03 (0.67~1.58) | 0.901 |  | 1.25 (0.87~1.8) | 0.230 |  | 1.21 (0.8~1.82) | 0.370 |  | 1.35 (0.89~2.06) | 0.155 |  | 1.07 (0.72~1.6) | 0.731 |  |  | |  |
|  | Q4 | 1 (0.64~1.57) | 0.986 |  | 1.08 (0.73~1.58) | 0.698 |  | 0.85 (0.56~1.3) | 0.455 |  | 1.25 (0.83~1.9) | 0.287 |  | 0.82 (0.54~1.26) | 0.372 |  |  | |  |
|  | Q5 | 1.16 (0.8~1.69) | 0.429 |  | 0.84 (0.56~1.25) | 0.379 |  | 1.06 (0.73~1.52) | 0.774 |  | 0.87 (0.6~1.25) | 0.445 |  | 1.15 (0.81~1.63) | 0.433 |  |  | |  |
| Female | Q1 | 1(Ref) |  |  | 1(Ref) |  |  | 1(Ref) |  |  | 1(Ref) |  |  | 1(Ref) |  |  |  | |  |
|  | Q2 | 0.8 (0.46~1.41) | 0.446 |  | 1.44 (0.8~2.56) | 0.222 |  | 0.61 (0.36~1.05) | 0.073 |  | 0.81 (0.46~1.4) | 0.446 |  | 1.61 (0.96~2.71) | 0.072 |  |  | |  |
|  | Q3 | 0.96 (0.55~1.67) | 0.879 |  | 1.52 (0.86~2.67) | 0.149 |  | 0.62 (0.34~1.11) | 0.106 |  | 0.89 (0.49~1.61) | 0.698 |  | 0.89 (0.5~1.57) | 0.678 |  |  | |  |
|  | Q4 | 0.83 (0.46~1.49) | 0.535 |  | 1.44 (0.82~2.54) | 0.203 |  | 0.49 (0.27~0.89) | 0.020 |  | 1.02 (0.54~1.93) | 0.940 |  | 1.08 (0.62~1.87) | 0.796 |  |  | |  |
|  | Q5 | 0.82 (0.46~1.46) | 0.500 |  | 1.22 (0.69~2.18) | 0.496 |  | 0.53 (0.29~0.94) | 0.031 |  | 0.88 (0.48~1.61) | 0.673 |  | 1.36 (0.79~2.33) | 0.264 |  |  | |  |
| Cigarette smoking |  |  |  | 0.141 |  |  | 0.783 |  |  | 0.834 |  |  | 0.124 |  |  | 0.412 |  | |  |
| Never | Q1 | 1(Ref) |  |  | 1(Ref) |  |  | 1(Ref) |  |  | 1(Ref) |  |  | 1(Ref) |  |  |  | |  |
|  | Q2 | 1.15 (0.7~1.89) | 0.572 |  | 1.05 (0.64~1.72) | 0.858 |  | 0.76 (0.47~1.24) | 0.273 |  | 0.76 (0.44~1.32) | 0.326 |  | 1.93 (1.18~3.15) | 0.009 |  |  | |  |
|  | Q3 | 1.13 (0.67~1.9) | 0.640 |  | 1.21 (0.75~1.96) | 0.425 |  | 0.88 (0.52~1.47) | 0.621 |  | 1.18 (0.69~2) | 0.548 |  | 1.18 (0.69~2.04) | 0.545 |  |  | |  |
|  | Q4 | 0.94 (0.54~1.63) | 0.817 |  | 1.32 (0.83~2.12) | 0.244 |  | 0.62 (0.36~1.06) | 0.080 |  | 1.29 (0.76~2.21) | 0.345 |  | 1.22 (0.71~2.1) | 0.461 |  |  | |  |
|  | Q5 | 1.13 (0.68~1.86) | 0.640 |  | 0.92 (0.56~1.52) | 0.744 |  | 0.87 (0.53~1.41) | 0.564 |  | 1.33 (0.83~2.13) | 0.239 |  | 1.64 (1~2.69) | 0.049 |  |  | |  |
| Current | Q1 | 1(Ref) |  |  | 1(Ref) |  |  | 1(Ref) |  |  | 1(Ref) |  |  | 1(Ref) |  |  |  | |  |
|  | Q2 | 0.67 (0.26~1.74) | 0.416 |  | 0.92 (0.41~2.08) | 0.844 |  | 0.47 (0.17~1.27) | 0.138 |  | 0.79 (0.31~2.04) | 0.623 |  | 0.48 (0.19~1.21) | 0.120 |  |  | |  |
|  | Q3 | 1.17 (0.48~2.84) | 0.731 |  | 0.97 (0.43~2.19) | 0.942 |  | 0.94 (0.37~2.41) | 0.896 |  | 0.87 (0.32~2.37) | 0.792 |  | 0.75 (0.33~1.73) | 0.500 |  |  | |  |
|  | Q4 | 0.83 (0.32~2.18) | 0.705 |  | 0.45 (0.16~1.28) | 0.135 |  | 0.49 (0.16~1.46) | 0.200 |  | 1.23 (0.46~3.31) | 0.681 |  | 0.44 (0.17~1.19) | 0.106 |  |  | |  |
|  | Q5 | 0.27 (0.08~0.89) | 0.031 |  | 0.68 (0.27~1.7) | 0.408 |  | 0.78 (0.3~2.02) | 0.607 |  | 0.44 (0.15~1.31) | 0.139 |  | 0.74 (0.33~1.65) | 0.457 |  |  | |  |
| Former | Q1 | 1(Ref) |  |  | 1(Ref) |  |  | 1(Ref) |  |  | 1(Ref) |  |  | 1(Ref) |  |  |  | |  |
|  | Q2 | 1.06 (0.67~1.67) | 0.797 |  | 1.46 (0.94~2.28) | 0.091 |  | 0.89 (0.56~1.42) | 0.629 |  | 1.06 (0.68~1.67) | 0.795 |  | 1.08 (0.7~1.66) | 0.719 |  |  | |  |
|  | Q3 | 1 (0.61~1.63) | 0.994 |  | 1.55 (1~2.39) | 0.049 |  | 1.17 (0.73~1.88) | 0.502 |  | 1.3 (0.81~2.07) | 0.279 |  | 0.91 (0.57~1.44) | 0.691 |  |  | |  |
|  | Q4 | 1.14 (0.7~1.85) | 0.607 |  | 1.29 (0.82~2.02) | 0.269 |  | 1.02 (0.64~1.62) | 0.940 |  | 1.28 (0.8~2.06) | 0.308 |  | 0.94 (0.6~1.48) | 0.791 |  |  | |  |
|  | Q5 | 1.3 (0.84~2) | 0.241 |  | 1.08 (0.68~1.7) | 0.749 |  | 0.94 (0.61~1.46) | 0.794 |  | 0.69 (0.43~1.1) | 0.120 |  | 1.12 (0.74~1.68) | 0.593 |  |  | |  |
| BMI, kg/m2 |  |  |  | 0.297 |  |  | 0.784 |  |  | 0.141 |  |  | 0.152 |  |  | 0.816 |  | |  |
| <25 | Q1 | 1(Ref) |  |  | 1(Ref) |  |  | 1(Ref) |  |  | 1(Ref) |  |  | 1(Ref) |  |  |  | |  |
|  | Q2 | 1.2 (0.66~2.16) | 0.552 |  | 1.37 (0.78~2.42) | 0.271 |  | 0.35 (0.18~0.66) | 0.001 |  | 0.49 (0.27~0.88) | 0.016 |  | 1.1 (0.65~1.87) | 0.727 |  |  | |  |
|  | Q3 | 1.09 (0.59~2.04) | 0.779 |  | 1.33 (0.75~2.33) | 0.329 |  | 0.58 (0.31~1.09) | 0.090 |  | 0.45 (0.23~0.86) | 0.015 |  | 0.96 (0.55~1.7) | 0.898 |  |  | |  |
|  | Q4 | 1.14 (0.6~2.19) | 0.684 |  | 1.19 (0.67~2.13) | 0.558 |  | 0.51 (0.28~0.96) | 0.037 |  | 0.7 (0.37~1.31) | 0.263 |  | 0.7 (0.37~1.3) | 0.260 |  |  | |  |
|  | Q5 | 0.75 (0.38~1.47) | 0.401 |  | 0.78 (0.41~1.48) | 0.439 |  | 0.71 (0.4~1.26) | 0.244 |  | 0.59 (0.32~1.1) | 0.095 |  | 1.03 (0.59~1.8) | 0.911 |  |  | |  |
| >=25 | Q1 | 1(Ref) |  |  | 1(Ref) |  |  | 1(Ref) |  |  | 1(Ref) |  |  | 1(Ref) |  |  |  | |  |
|  | Q2 | 0.95 (0.65~1.4) | 0.809 |  | 1.15 (0.8~1.66) | 0.455 |  | 1.03 (0.71~1.5) | 0.872 |  | 1.15 (0.77~1.73) | 0.487 |  | 1.35 (0.94~1.94) | 0.108 |  |  | |  |
|  | Q3 | 1.05 (0.71~1.56) | 0.799 |  | 1.26 (0.88~1.8) | 0.211 |  | 1.23 (0.83~1.82) | 0.292 |  | 1.69 (1.14~2.53) | 0.010 |  | 0.98 (0.66~1.46) | 0.929 |  |  | |  |
|  | Q4 | 0.91 (0.6~1.38) | 0.665 |  | 1.2 (0.84~1.72) | 0.326 |  | 0.89 (0.6~1.34) | 0.585 |  | 1.59 (1.06~2.39) | 0.025 |  | 1.07 (0.73~1.58) | 0.714 |  |  | |  |
|  | Q5 | 1.16 (0.81~1.66) | 0.428 |  | 0.97 (0.67~1.4) | 0.854 |  | 0.89 (0.61~1.3) | 0.558 |  | 1.07 (0.74~1.56) | 0.713 |  | 1.3 (0.92~1.84) | 0.141 |  |  | |  |
| HR, hazard ratio; CI, confidence interval; BMI, body mass index. Adjusted for age (continuous), sex (male vs. female), trial arm (intervention vs. control), race (white, non-Hispanic vs. black, non-Hispanic vs. Hispanic vs. others), marital status (married vs. unmarried), education level (≤high school vs. ≥some college), aspirin use (no vs. yes), diabetes (no vs. yes), cigarette smoking (never vs. current vs. former), BMI (<25kg/m2 vs. ≥25kg/m2), family history of colorectal cancer (yes vs. no vs. possibly), alcohol drinking (never vs. former vs. current), history of colorectal polyps (no vs. yes), history of colon comorbidities (no vs. yes), and energy from diet (continuous). In subgroup analyses stratified by age, sex, trial arm, aspirin use, cigarette smoking, BMI, alcohol drinking, family history of colorectal cancer, history of colorectal polyps, history of colon comorbidities, and diabetes, HRs were adjusted except for the stratification factor. *P*-value for interaction was calculated by the like-hood ratio test, and *P*>0.05 was considered no statistical significance. | | | | | | | | | | | | | | | | | |  | |
|  |  |  |  |  |  |  |  |  |  |  |  |  |  |  |  |  |  |  | |
|  |  |  |  |  |  |  |  |  |  |  |  |  |  |  |  |  |  |  | |
|  |  |  |  |  |  |  |  |  |  |  |  |  |  |  |  |  |  |  | |
|  |  |  |  |  |  |  |  |  |  |  |  |  |  |  |  |  |  |  | |
|  |  |  |  |  |  |  |  |  |  |  |  |  |  |  |  |  |  |  | |
|  |  |  |  |  |  |  |  |  |  |  |  |  |  |  |  |  |  |  | |
|  |  |  |  |  |  |  |  |  |  |  |  |  |  |  |  |  |  | |  |

**Supplementary Table S8. Sensitivity analyses on the association between energy-adjusted tomato-related products/lycopene intakes and colorectal cancer mortality.**

|  | Primary analysis | Adjusted hazard ratio (95% confidence interval) ^a^ | | | | |  |
| --- | --- | --- | --- | --- | --- | --- | --- |
|  |  | Excluding events within the first 2 years of follow-up (excluded 1241 participants) | Excluding events with extreme values of energy intake (excluded 3608 participants) | Additional Model 1 | Additional Model 2 | Additional Model 3 | Additional Model 4 |
| Raw tomato (g/day) |  |  |  |  |  |  |  |
| Q1 | 1(Ref) | 1(Ref) | 1(Ref) | 1(Ref) | 1(Ref) | 1(Ref) | 1(Ref) |
| Q2 | 1.18 (0.87~1.6) | 1.18 (0.86~1.6) | 1.12 (0.82~1.54) | 1.18 (0.87~1.61) | 1.18 (0.87~1.6) | 1.17 (0.86~1.6) | 1.18 (0.87~1.6) |
| Q3 | 1.28 (0.95~1.73) | 1.27 (0.94~1.71) | 1.24 (0.92~1.69) | 1.28 (0.95~1.73) | 1.28 (0.95~1.73) | 1.28 (0.95~1.73) | 1.29 (0.95~1.75) |
| Q4 | 1.15 (0.85~1.55) | 1.07 (0.78~1.46) | 1.06 (0.77~1.46) | 1.15 (0.84~1.57) | 1.14 (0.83~1.56) | 1.14 (0.84~1.56) | 1.15 (0.84~1.58) |
| Q5 | 0.93 (0.68~1.28) | 0.92 (0.67~1.27) | 0.92 (0.67~1.28) | 0.93 (0.67~1.31) | 0.93 (0.66~1.3) | 0.92 (0.66~1.3) | 0.93 (0.66~1.31) |
| *P* for trend | 0.648 | 0.484 | 0.577 | 0.724 | 0.681 | 0.679 | 0.728 |
| continuous (log2) | 1 (0.94~1.06) | 1 (0.94~1.07) | 0.99 (0.93~1.06) | 1 (0.93~1.07) | 1 (0.93~1.07) | 1 (0.93~1.07) | 1 (0.93~1.07) |
| Tomato salsa (g/day) |  |  |  |  |  |  |  |
| Q1 | 1(Ref) | 1(Ref) | 1(Ref) | 1(Ref) | 1(Ref) | 1(Ref) | 1(Ref) |
| Q2 | 0.8 (0.58~1.1) | 0.81 (0.59~1.12) | 0.71 (0.51~0.99) | 0.8 (0.58~1.1) | 0.79 (0.57~1.09) | 0.79 (0.57~1.09) | 0.8 (0.58~1.1) |
| Q3 | 1.08 (0.78~1.5) | 1.12 (0.8~1.56) | 0.91 (0.65~1.29) | 1.05 (0.76~1.46) | 1.01 (0.73~1.42) | 1.02 (0.73~1.42) | 1.02 (0.73~1.43) |
| Q4 | 0.84 (0.6~1.17) | 0.79 (0.56~1.12) | 0.65 (0.46~0.93) | 0.82 (0.59~1.15) | 0.8 (0.57~1.12) | 0.8 (0.57~1.13) | 0.81 (0.57~1.14) |
| Q5 | 0.98 (0.72~1.33) | 0.98 (0.72~1.34) | 0.84 (0.6~1.16) | 0.97 (0.7~1.32) | 0.94 (0.69~1.29) | 0.95 (0.69~1.31) | 0.96 (0.7~1.31) |
| *P* for trend | 0.97 | 0.886 | 0.302 | 0.926 | 0.791 | 0.851 | 0.867 |
| continuous (log2) | 1.03 (0.98~1.09) | 1.03 (0.98~1.09) | 1.03 (0.97~1.09) | 1.03 (0.98~1.09) | 1.03 (0.98~1.09) | 1.03 (0.98~1.09) | 1.03 (0.98~1.09) |
| Tomato juice (g/day) |  |  |  |  |  |  |  |
| Q1 | 1(Ref) | 1(Ref) | 1(Ref) | 1(Ref) | 1(Ref) | 1(Ref) | 1(Ref) |
| Q2 | 1.05 (0.77~1.44) | 1.09 (0.79~1.5) | 1.02 (0.73~1.41) | 1.07 (0.78~1.46) | 1.06 (0.77~1.45) | 1.06 (0.77~1.45) | 1.06 (0.77~1.45) |
| Q3 | 1.09 (0.78~1.51) | 1.14 (0.82~1.59) | 1.01 (0.72~1.42) | 1.1 (0.79~1.53) | 1.07 (0.77~1.5) | 1.08 (0.77~1.5) | 1.08 (0.77~1.5) |
| Q4 | 1.02 (0.73~1.44) | 1.07 (0.76~1.52) | 0.93 (0.65~1.33) | 1.02 (0.72~1.44) | 0.99 (0.7~1.4) | 0.99 (0.7~1.4) | 0.99 (0.7~1.4) |
| Q5 | 1.08 (0.79~1.47) | 1.07 (0.78~1.47) | 0.97 (0.7~1.35) | 1.1 (0.79~1.52) | 1.05 (0.75~1.46) | 1.05 (0.75~1.46) | 1.04 (0.75~1.45) |
| *P* for trend | 0.746 | 0.821 | 0.698 | 0.726 | 0.966 | 0.96 | 0.987 |
| continuous (log2) | 1.03 (0.94~1.13) | 1.04 (0.94~1.14) | 1.03 (0.93~1.13) | 1.03 (0.93~1.14) | 1.02 (0.92~1.13) | 1.03 (0.93~1.14) | 1.03 (0.93~1.14) |
| Tomato catsup (g/day) |  |  |  |  |  |  |  |
| Q1 | 1(Ref) | 1(Ref) | 1(Ref) | 1(Ref) | 1(Ref) | 1(Ref) | 1(Ref) |
| Q2 | 0.9 (0.65~1.25) | 0.93 (0.67~1.3) | 0.85 (0.61~1.2) | 0.9 (0.65~1.24) | 0.9 (0.65~1.25) | 0.9 (0.65~1.25) | 0.89 (0.64~1.24) |
| Q3 | 1.2 (0.86~1.67) | 1.26 (0.9~1.76) | 1.03 (0.73~1.47) | 1.16 (0.83~1.62) | 1.15 (0.82~1.6) | 1.15 (0.82~1.6) | 1.14 (0.81~1.59) |
| Q4 | 1.29 (0.93~1.8) | 1.36 (0.97~1.91) | 1.16 (0.81~1.65) | 1.22 (0.87~1.7) | 1.2 (0.85~1.69) | 1.19 (0.85~1.68) | 1.18 (0.83~1.66) |
| Q5 | 0.92 (0.67~1.26) | 0.96 (0.7~1.32) | 0.86 (0.62~1.2) | 0.88 (0.64~1.21) | 0.89 (0.65~1.22) | 0.88 (0.64~1.21) | 0.87 (0.63~1.19) |
| *P* for trend | 0.757 | 0.581 | 0.925 | 0.906 | 0.924 | 0.866 | 0.774 |
| continuous (log2) | 1 (0.94~1.07) | 1 (0.94~1.07) | 0.99 (0.93~1.06) | 0.99 (0.92~1.05) | 0.99 (0.92~1.05) | 0.98 (0.92~1.05) | 0.98 (0.92~1.04) |
| Lycopene (mcg/day) |  |  |  |  |  |  |  |
| Q1 | 1(Ref) | 1(Ref) | 1(Ref) | 1(Ref) | 1(Ref) | 1(Ref) | 1(Ref) |
| Q2 | 1.26 (0.94~1.7) | 1.32 (0.98~1.78) | 1.22 (0.9~1.66) | 1.28 (0.94~1.73) | 1.28 (0.94~1.74) | 1.28 (0.94~1.73) | 1.28 (0.94~1.73) |
| Q3 | 1 (0.72~1.37) | 0.98 (0.7~1.36) | 0.95 (0.68~1.32) | 0.99 (0.71~1.39) | 0.99 (0.71~1.39) | 0.99 (0.71~1.38) | 0.99 (0.71~1.38) |
| Q4 | 0.97 (0.71~1.35) | 1 (0.72~1.38) | 0.87 (0.62~1.22) | 0.97 (0.69~1.36) | 0.97 (0.69~1.36) | 0.96 (0.68~1.35) | 0.96 (0.68~1.35) |
| Q5 | 1.26 (0.94~1.68) | 1.23 (0.91~1.65) | 1.19 (0.88~1.61) | 1.29 (0.93~1.79) | 1.27 (0.91~1.77) | 1.26 (0.9~1.76) | 1.25 (0.9~1.75) |
| *P* for trend | 0.495 | 0.672 | 0.948 | 0.565 | 0.635 | 0.692 | 0.706 |
| continuous (log2) | 0.99 (0.9~1.09) | 0.98 (0.89~1.07) | 0.96 (0.87~1.06) | 0.99 (0.89~1.11) | 0.99 (0.89~1.1) | 0.98 (0.88~1.1) | 0.98 (0.88~1.09) |

Extreme values of energy intake were included <800 or >4000 kcal/day for males and <500 or >3500 kcal/day for females.

Additional Model 1: adjusted for the factors listed in the fully-adjusted model (table 2, model 3), and red meat (g/day), processed meat (g/day), fruit (g/day), vegetables (g/day), whole grain (servings/day), dairy (servings/day), sugar (tsp/day), and dietary fiber (g/day).

Additional Model 2: adjusted for additional Model 1 plus glycemic load, glycemic index, protein (% energy), total fat (% energy), and carbohydrate (% energy).

Additional Model 3: adjusted for additional Model 2 plus total calcium (mg/day), folate (mg/day), magnesium (mg/day), iron (mg/day), and vitamin D (mcg/day).

Additional Model 4: adjusted additional Model 3, and further adjusted olive oil (g/day). All covariates above-mentioned were treated as continuous variables in the models.
